# A heme pocket aromatic quadrupole modulates gas binding to cytochrome *c*′-β: Implications for NO sensors

**DOI:** 10.1016/j.jbc.2023.104742

**Published:** 2023-04-24

**Authors:** Hannah R. Adams, Dimitri A. Svistunenko, Michael T. Wilson, Sotaro Fujii, Richard W. Strange, Zoe A. Hardy, Priscilla A. Vazquez, Tyler Dabritz, Gabriel J. Streblow, Colin R. Andrew, Michael A. Hough

**Affiliations:** 1 School of Life Sciences, University of Essex, Colchester, Essex, United Kingdom; 2 Graduate School of Biosphere Science, Hiroshima University, Higashi-Hiroshima, Hiroshima, Japan; 3 Diamond Light Source Ltd, Harwell Science and Innovation Campus, Didcot, United Kingdom; 4 Department of Chemistry & Biochemistry, Eastern Oregon University, La Grande OR, USA

**Keywords:** aromatic quadrupole, carbon monoxide, cytochrome, nitric oxide

## Abstract

The structural basis by which gas-binding heme proteins control their interactions with NO, CO, and O_2_ is fundamental to enzymology, biotechnology, and human health. Cytochromes *c*′ (cyts *c′*) are a group of putative NO-binding heme proteins that fall into two families: the well-characterized four alpha helix bundle fold (cyts *c′*-α) and an unrelated family with a large beta-sheet fold (cyts *c′*-β) resembling that of cytochromes P460. A recent structure of cyt *c′*-β from *Methylococcus capsulatus* Bath revealed two heme pocket phenylalanine residues (Phe 32 and Phe 61) positioned near the distal gas-binding site. This feature, dubbed the “Phe cap,” is highly conserved within the sequences of other cyts *c′*-β but is absent in their close homologs, the hydroxylamine-oxidizing cytochromes P460, although some do contain a single Phe residue. Here, we report an integrated structural, spectroscopic, and kinetic characterization of cyt *c*′-β from *Methylococcus capsulatus* Bath complexes with diatomic gases, focusing on the interaction of the Phe cap with NO and CO. Significantly, crystallographic and resonance Raman data show that orientation of the electron-rich aromatic ring face of Phe 32 toward distally bound NO or CO is associated with weakened backbonding and higher off rates. Moreover, we propose that an aromatic quadrupole also contributes to the unusually weak backbonding reported for some heme-based gas sensors, including the mammalian NO sensor, soluble guanylate cyclase. Collectively, this study sheds light on the influence of highly conserved distal Phe residues on heme–gas complexes of cytochrome *c*’-β, including the potential for aromatic quadrupoles to modulate NO and CO binding in other heme proteins.

The structural features that enable heme protein active sites to recognize and control their reactivity with exogenous ligands are of fundamental interest for enzymology, biotechnology, and human health. Many ammonia-oxidizing and methane-oxidizing bacteria contain related ligand-binding cytochromes with a shared but unusual β-sheet fold. Of these, cytochrome P460 (cytP460) is a heme enzyme that oxidizes hydroxylamine to N_2_O as part of nitrification (1), whereas the evolutionarily related cytochrome *c*′-β (cyt *c*′-β) binds the diatomic gases, NO and CO (2). Proposed roles for cyt *c*′-β proteins include protection against NO stress generated during nitrification (3). Key to the functional evolution of cyt *c*′-β from cytP460 is the distal heme pocket environment. Catalytically active cytsP460 have ionizable side chains within the distal heme pocket as well as an unusual Lys–porphyrin crosslink. By contrast, our recent crystal structure of cyt *c*′-β from *Methylococcus capsulatus* (Bath) (McCP-β) (4) reveals a hydrophobic distal pocket dominated by two Phe residues (F32 and F61), a structural feature we have named the “Phe cap.” In cytsP460, F61 is replaced by the crosslinking Lys residue whilst F32 is generally replaced with residues with more ionizable side chains, although a few examples, such as the inactive cytP460 variant from *Nitrosomonas* sp. AL212 (5), do have a Phe in this position. Amino acid sequence alignments suggest that a Phe cap is conserved in many other cyts *c′*-β (6). However, the function of the Phe cap, particularly its influence on heme–gas coordination, has not been investigated.

Several other types of gas-binding heme proteins also contain noncoordinating aromatic side chains (Phe, Trp, or Tyr) near the distal gas-binding site. In some heme proteins, these bulky aromatic groups provide steric hindrance to ligand binding. For example, the distal binding sites of alpha helical cytochromes *c′*-α (cyts *c′*-α)—which are unrelated to cyts *c′*-β—contain a bulky aromatic (Phe, Tyr) or aliphatic (Leu, Met) residue close to the distal heme site (7). As well as creating a hydrophobic microenvironment that excludes water and ionic species, the occluding aromatic (or aliphatic) group sterically hinders distal heme coordination, lowering the on-rates for diatomic gas binding, and triggering an unusual distal to proximal heme–NO switch in which a six-coordinate ferrous NO (6cNO) precursor converts to a five-coordinate complex (5cNO) on the opposite (proximal) heme face *via* a transient dinitrosyl species (7, 8, 9, 10, 11, 12). The formation of proximal 5cNO species has generated considerable interest as a novel means of regulating heme–NO affinity, including in heme-based gas sensors (13).

Nonpolar aromatic side chains are also found within the distal heme pockets of NO-sensing gas proteins that contain a heme nitric oxide oxygen (H-NOX) domain, including the mammalian NO-sensor soluble guanylate cyclase (sGC) (Phe 74 residue) and a bacterial analog, *Nostoc* sp. H-NOX (*Ns* H-NOX) (Trp 74 residue) (14, 15). Unlike the sterically constrained distal heme sites of cyts *c′*-α, NO and CO binding to sGC and *Ns* H-NOX is quite rapid (16, 17), signifying that the distal pocket aromatic residues in these proteins provide minimal steric constraints to heme–gas coordination. Both sGC and *Ns* H-NOX selectively bind NO (rather than O_2_) within an aerobic environment, and it has been proposed that their distal pocket aromatic rings help discriminate against O_2_ by creating a hydrophobic environment, devoid of H-bond donors that could stabilize the highly polar Fe(II)O_2_ unit (18).

An additional property of aromatic rings is the existence of an electric quadrupole. In the case of nonpolar aromatics (which have no overall permanent dipole), local regions of partial negative charge exist with the electron-rich π system above and below the aromatic ring, counteracted by partial positive charge on the ring carbons (19). Aromatic quadrupoles play important roles in stabilizing protein and nucleic acid structures through π–π stacking of multiple aromatic rings as well as cation–π interactions and can also contribute to molecular recognition of substrates (20). However, the influence of aromatic quadrupoles in modulating ligand binding within the active sites of heme proteins, including gas-binding and sensing proteins, has not been reported. In this integrated crystallographic, spectroscopic, and kinetic study, we describe the interaction of McCP-β with diatomic gases, revealing a quadrupole interaction between a Phe aromatic ring and distally bound NO and CO ligands. Our findings reveal a novel determinant of heme–gas reactivity, with possible implications for other heme proteins, including heme-based NO sensors in animals and bacteria.

## Results

### Crystal structures of CO and NO complexes of wt McCP-β: interactions with the Phe cap

**Figure 1:**
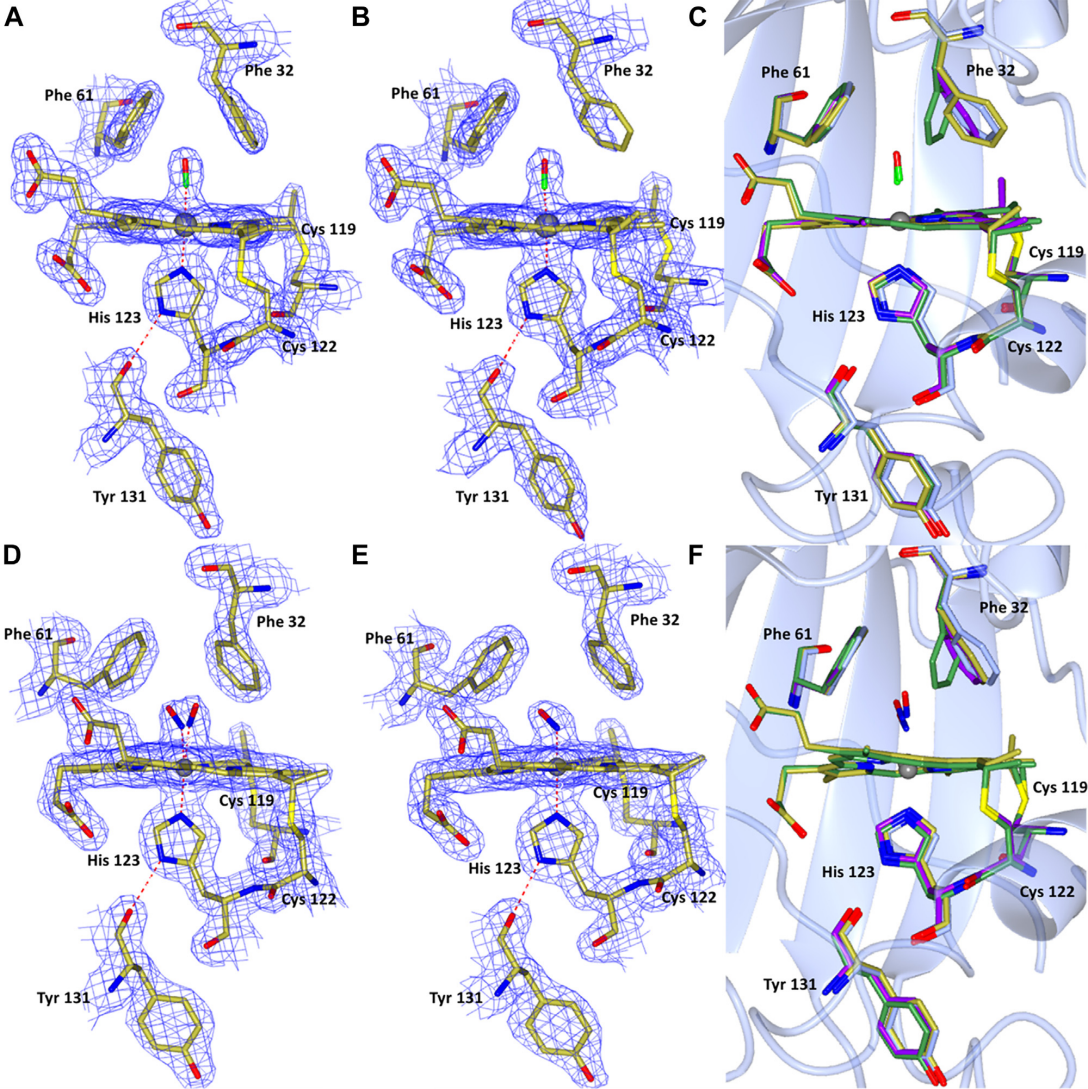
**Hemes A and B of McCP-β**. With either CO (*A* and *B*) or NO bound (*D* and *E*). Only one orientation of CO is seen with an angle of 174/173°. Two orientations of NO are seen in monomer A and one in monomer B. The aromatic ring of Phe 32 can be seen to be rotated away from the CO molecule in the second heme. Comparison of Fe(II) McCP-β (heme A in *blue* and heme B in *green*), and both hemes A (*purple*) and B (*gold*) with CO (*C*) or NO (*F*) bound, Phe 32 can be seen to move upon introduction of a ligand to the distal side of the heme. McCP-β, cyt *c*′-β from *Methylococcus capsulatus* (Bath).

Crystal structures of Fe(II) McCP-β soaked with CO and NO were determined to 1.6 Å and 1.56 Å, respectively (Fig. 1 and Table 1),and compared with the chemically reduced Fe(II) McCP-β structure in the absence of ligands, determined at 1.68 Å (Fig. 1 and Table S1). The ligand-free Fe(II) structure resembles that of previously reported as isolated McCP-β crystallized in the Fe(III) state (Fig. S1) (4). However, it is well known that the X-ray beam used for data collection can readily reduce heme protein crystals from Fe(III) to Fe(II) (21). The homodimeric and tertiary structures of the Fe(II), Fe(II)CO, and Fe(II)NO McCP-β complexes resemble those of ligand-free protein (4), with significant structural changes being essentially limited to the heme distal pocket (Fig. 1).

**Table 1:** Data collection, processing, and refinement statistics

Dataset	MCCP-CO	MCCP-NO	F32V-CO	F32V-NO	F61V-CO	F61V-NO
Resolution (Å)	47.2–1.60	47.25–1.56	52.94–1.77	61.16–1.94	52.72–2.09	33.59–1.68
Unit cell, (Å)	a = b = c= 105.5	a = b = c = 105.7	a = b = c = 105.9	a = b = c = 105.9	a = b = c = 105.5	a = b = c = 106.2
Unique reflections	51,778 (2607)	56,066 (2797)	38,716 (1958)	29,544 (1570)	23,393 (1514)	45,287 (2318)
Completeness (%)	100 (100)	100 (100)	100 (100)	100 (100)	100 (100)	99 (100)
Redundancy	20 (21)	20 (21)	6.7 (6.8)	10 (11)	19 (21)	3 (3)
*R*_meas_	0.069 (2.73)	0.080 (2.52)	0.069 (2.04)	0.044 (2.94)	0.032 (5.27)	0.049 (0.91)
I/σ(I)	26.3 (1.4)	20.0 (1.5)	18.7 (0.6)	13.5 (0.5)	22.7 (0.6)	11.7 (1.1)
CC_1/2_	1.0 (0.5)	1.0 (0.5)	1.0 (0.3)	1.0 (0.3)	1.0 (0.4)	1.0 (0.6)
Wilson *B*-factor (Å^2^)	25.9	26.0	32.5	39.0	45.31	29.8
*R*_work_	0.181	0.181	0.191	0.187	0.204	0.178
*R*_free_	0.198	0.202	0.212	0.207	0.269	0.202
RMSD bond length (°)	0.014	0.015	0.015	0.013	0.013	0.015
RMSD bond angles (Å)	2.2	1.86	2.09	2.21	2.20	1.86
Ramachandran favored (%)	96.3	95.0	95.9	95.9	94.0	98.1
Protein Data Bank accession code	6ZSK	7ZPS	7ZSX	7ZSW	7ZTI	7ZQZ

In both the Fe(II)CO and Fe(II)NO crystal structures, the heme Fe centers are six coordinate and can therefore be referred to as 6cCO and 6cNO, respectively, with the gas ligand bound on the distal side opposite the proximal His 123 ligand (Table 2). For the Fe(II)CO structure, the homodimer exhibits Fe–C distances of 1.78 and 1.86 Å, and near-linear Fe–C–O angles of 173° and 174° for monomer A and B, respectively (Fig. 1, *A* and *B*). In the case of the Fe(II)NO structure, two orientations of distal NO with partial occupancy were modeled bound to the heme in monomer A (Fig. 1*D*). One NO (occupancy = 0.5) is orientated toward Leu 28 with an Fe–N distance of 2.01 Å and an Fe–N–O angle of 148°, whereas the other (occupancy = 0.5) is orientated in the opposite direction toward Gly 82 with an Fe–N distance of 1.87 Å and an Fe–N–O angle of 136°. Only one orientation of NO is seen in monomer B (Fig. 1*E*) (toward Gly 82) with an Fe–N distance of 1.80 Å and an Fe–N–O angle of 122°.

**Table 2:** Heme site parameters in McCP-β, F32V, and F61V crystal structures

Structure	Fe–His N (Å)	Fe–XO (Å)	Fe–X–O (°)
Fe(III) McCP (6HIH)	2.10/2.09	**—**	**—**
Fe(III) F32V	2.00/2.08	**—**	**—**
Fe(III) F61V	2.02/2.05	**—**	**—**
Fe(II) McCP	2.11/2.13	—	—
Fe(II) F32V	2.07/2.07	—	—
Fe(II) F61V	2.10/2.13	—	—
McCP–NO	2.18/2.20	2.01/1.87/1.80	148/136/122
F32V–NO	2.53/2.62	1.90/1.97	110/92
F61V–NO	2.93/3.03	1.86/1.86	130/135
McCP–CO	2.08/2.05	1.86/1.78	174/173
F32V–CO	2.03/2.07	2.03/2.07	164/165
F61V–CO	2.10/2.09	2.01/2.00	166/177

The crystal structures shed light on the interaction of the distal Phe cap with exogenous gas ligands. Unlike Phe 61, which shows no significant conformational changes upon NO or CO coordination, Phe 32 can be seen to rotate around the Cβ–Cγ bond, allowing access to the distal Fe-binding site and moving its aromatic ring face away from the heme (Fig. 1, *C* and *F*). In the NO-bound structures, the rotation around the Phe 32 Cβ–Cγ bond in comparison to the Fe(II) structure is 6° and 15° in hemes A and B, respectively, such that the aromatic ring face of Phe 32 presents toward the NO ligand and the ring atoms are 3.5 to 3.6 Å from the NO ligand. In heme A, Phe 32 also moves sideways away from the heme by 1.2 Å in comparison to the Fe(II) structure, no similar movement is seen in heme B. Within the Fe(II)CO structure, rotation around the Phe 32 Cβ–Cγ bond appears greater in one of the monomers resulting in two distal pocket conformations. In monomer A, the Phe 32 ring face is rotated 30° away from the CO ligand with a distance of 4.5 Å between the CO ligand and the ring atoms of Phe 32. By contrast, in monomer B, the aromatic ring is presented toward the CO molecule (which more closely resembles the positioning seen in the NO complex) with a rotation of around 19° and a distance of 3.4 Å between the CO ligand and the Phe 32 ring atoms. Phe 61, which does not change its conformation, has its Cε2 atom some 3.2 Å from the O of the CO ligand, but other ring atoms as far as 3.9 Å away, consistent with a more offset position in both monomers.

### Spectroscopic properties of CO and NO complexes of wt McCP-β: evidence for a negatively polarized gas-binding environment

**Figure 2:**
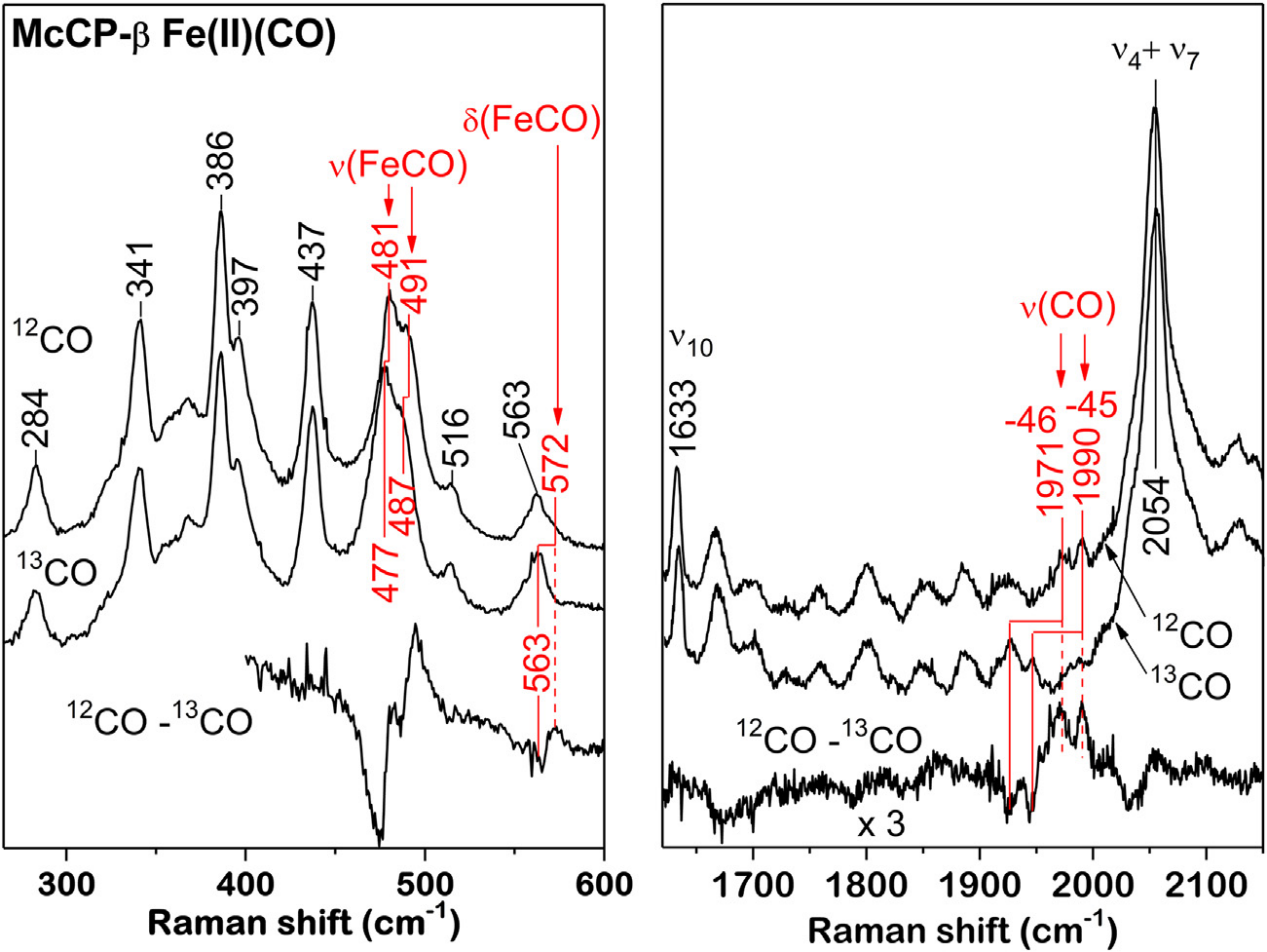
**Room temperature resonance Raman spectra of 6cCO McCP-β complexes prepared with**^**12**^**CO and**^**13**^**CO recorded in the low frequency (*left panel*) and high frequency (*right panel*) regions, together with**^**12**^**CO to**^**13**^**CO difference spectra.** McCP-β, cyt *c*′-β from *Methylococcus capsulatus* (Bath);

Spectroscopic measurements (UV–visible absorption, resonance Raman [RR], and electron paramagnetic resonance [EPR]) provide complementary structural information on the interaction of McCP-β with diatomic gases in solution. In line with previous absorption data (2), Fe(II) McCP-β (λ_max_ = 431 and 552 nm) reacts with CO to form a 6c Fe(II)CO complex (6cCO) (λ_max_ = 418, 533, and 560 nm). RR measurements using relatively low laser powers to minimize photodissociation of the CO ligand reveal RR features typical of a 6cCO complex, including porphyrin marker RR bands: ν_4_ (1373 cm^−1^), ν_2_ (1591 cm^−1^), and ν_10_ (1633 cm^−1^) (Fig. S2). Substitution with ^13^CO identifies a pair of ν(FeCO) vibrations at 481 and 491 cm^−1^ with 4 cm^−1^ downshifts, together with a weaker δ(FeCO) mode at 572 cm^−1^ with a downshift of ∼9 cm^−1^ (Fig. 2). Although only weakly enhanced, a pair of ν(CO) vibrations is also identified at 1971 and 1990 cm^−1^ with downshifts of ∼46 and ∼45 cm^−1^, respectively (Fig. 2). The existence of doublets for both the ν(FeCO) and ν(CO) modes is consistent with two distinct heme–CO environments that impact the degree of Fe(II)→CO(π∗) backbonding.

**Figure 3:**
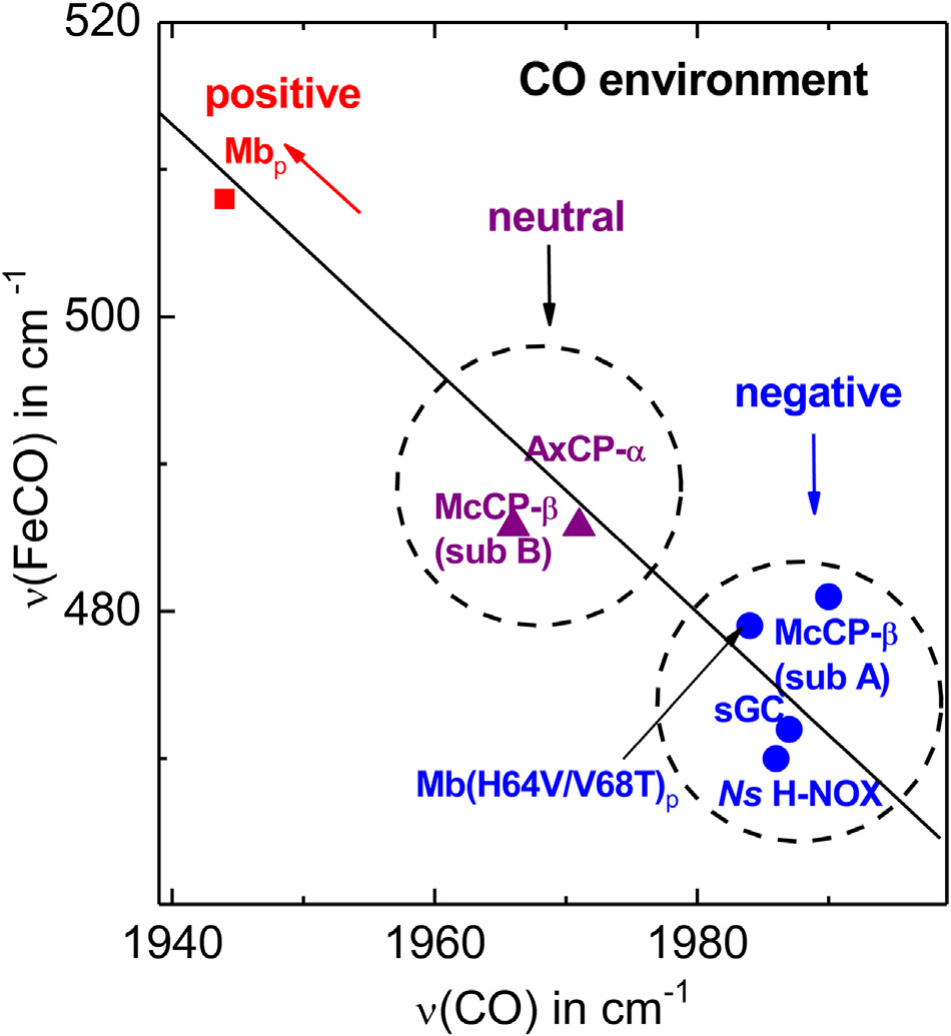
**Relationship between ν(FeCO) and ν(CO) frequencies in 6cCO heme complexes, including data for McCP-β.** Vibrational data are taken from Table 3. The *solid line* is the reported empirical relationship between the ν(FeCO) and ν(CO) frequencies of model porphyrins (arising from variations in Fe(II)→CO(π∗) backbonding) (22). Heme protein 6cCO complexes exhibit a vibrational trend similar to that of model complexes, which allows ν(FeCO) and ν(CO) frequencies to report on the heme pocket polarity. McCP-β, cyt *c*′-β from *Methylococcus capsulatus* (Bath).

Protein environments that facilitate Fe(II)→CO(π∗) backbonding (and the associated transfer of electron density to the CO ligand) increase the ν(FeCO) frequency and lower the ν(CO) frequency (22). Conversely, environments that inhibit backbonding result in low ν(FeCO) frequencies and high ν(CO) frequencies. Indeed, the type of heme pocket environment can be gauged from the inverse correlation of ν(FeCO) *versus* ν(CO) frequencies (Fig. 3) (22). In the case of McCP-β, the ν(CO)/ν(FeCO) frequency pair of 1971/491 cm^−1^ is typical of neutral nonpolar heme pockets, such as AxCP-α (1966/491 cm^−1^) and other cyt-*c*′-α proteins (Fig. 3 and Table 3) (23). On the other hand, the 1990/481 cm^−1^ frequency pair signifies weaker Fe(II)→CO(π∗) backbonding, consistent with an electron-rich environment (Fig. 3 and Table 3).

**Table 3:** Influence of heme pocket microenvironments on the ν(FeCO) and ν(CO) vibrational frequencies (cm^−1^) of 6cCO heme protein complexes

6cCO complex	ν(FeCO)	ν(CO)	Polarity of CO microenvironment^a^	Protein Data Bank code	Reference
McCP-β wt (subunit A)	481	1990	Negative (F32 aromatic quadrupole)	6ZSK	tw
McCP-β wt (subunit B)	491	1971	Neutral	6ZSK	tw
McCP-β F61V	483		Negative (F32 aromatic quadrupole)	7ZTI	tw
McCP-β F32V	497		Neutral	7ZSX	tw
AxCP-α	491	1966	Neutral	2YLD	(9, 23)
pig Mb wt	508	1944	Positive (H64 H-bond)	1MWC	(25, 26, 52)
pig Mb (V68T)	496	1961	Positive (H64) and negative (T68 hydroxy lone pair)	1YCB	(25, 26)
pig Mb (H64V/V68T)	479	1984	Negative (T68 hydroxy lone pair)		(25, 26)
*Ns* H-NOX	470	1986	Negative (W74 aromatic quadrupole)	2O0G	(14, 53)
sGC	472	1987	Negative (F74 aromatic quadrupole)	6JT2	(15, 54, 55)

Abbreviations: tw, this work.

a For McCP-β, AxCP-α, and *Ns* H-NOX, the polarity ascribed to the microenvironment of the CO ligand is based on crystallographic data of the 6cCO complexes as well as frequencies of the ν(FeCO) and ν(CO) RR modes, which report the degree of Fe(II)→CO(π∗) backbonding. Although the location of gas ligands in sGC has not been determined crystallographically, cryo-electron microscopy indicates that the negatively polarized F74 ring face is positioned to interact with distally bound gases. For the V68T single variant of pig Mb, the crystal structure shows that the CO ligand experiences both positive polarity (H-bond from His64) and negative polarity (lone pair from the T68 hydroxy lone pair). For the Mb H64V/V68T double variant, it is assumed that only the negative polarity from T68 remains (consistent with its relatively high ν(CO) frequency and relatively low ν(FeCO) frequency).

RR evidence for two types of Fe–CO heme environment in McCP-β is consistent with the crystal structure described previously (Fig. 1, *A* and *B*). In heme A, the plane of the Phe 32 capping residue is directed toward the CO oxygen (3.4 Å distant), whereas in heme B, Phe 32 is rotated so that its closest atom is 4.5 Å from CO and it no longer presents the plane. We propose that the local negative polarization resulting from the aromatic quadrupole of the Phe 32 π-face inhibits Fe(II)→CO(π∗) backbonding when oriented toward the CO, leading to an unusually high ν(CO) frequency and low ν(FeCO). Conversely, when the Phe 32 ring face is rotated away from CO, backbonding interactions and ν(CO)/ν(FeCO) frequencies resemble those of normal (nonpolarized) hydrophobic environments. Although distal Phe residues exist within members of the cyt *c*′-α protein family (*e.g.*, RcCP-α, SfCP-α, and the L16F variant of AxCP-α), there are no crystal structures available for their Fe(II)CO states. RR spectra of the RcCP–α Fe(II)CO complex reveal ν(CO) and ν(FeCO) frequencies consistent with a single nonpolarized environment, suggesting that CO does not interact with the Phe aromatic dipole (24). For SfCP-α and L16F AxCP-α, extremely low heme–CO affinities (attributed to distal steric constraints) prevented RR characterization of their 6cCO complexes (11, 12).

To our knowledge, McCP-β would be the first example of a heme protein in which an aromatic π-electron quadrupole influences Fe(II)→CO(π∗) backbonding. The only other structurally characterized Fe(II)CO complex in which local negative polarity influences backbonding (albeit from a lone pair of electrons) involves the V68T mutation of pig myoglobin (Mb). The crystal structure of the V68T variant Fe(II)CO complex shows the CO ligand interacting with a lone pair of electrons from the threonine hydroxy group, some 3.16 Å distant (25). Corresponding RR spectra reveal a 12 cm^−1^ decrease in ν(FeCO) and a 17 cm^−1^ increase in ν(CO) relative to wt Mb (Table 3) (25, 26), consistent with weakened backbonding. A similar interaction presumably occurs in the H64V/V68T Mb double variant (25, 26), where the removal of positive polarity from the His64 H-bond donor further lowers ν(FeCO), while boosting ν(CO) (Table 3). Crystallographic and RR data from the minihemoglobin from *Cerebratulus lacteus* (CerHb) also suggest that a nonbonded electron pair in the distal pocket (arising from the phenolic oxygen of TyrB10) might inhibit Fe(II)→CO(π∗) backbonding (27), although its influence is complicated by adjacent H-bond donors.

In contrast to the Fe(II)NO McCP-β crystal structure, which shows only a 6c adduct, UV–visible absorbance measurements of the Fe(II)NO complex in solution reveal a pH–dependent equilibrium between a 6c Fe(II)NO species (6cNO, λ_max_ ∼416 nm) (favored at high pH) and a 5c Fe(II)NO species in which the His ligand dissociates (5cNO, λ_max_ ∼395 nm) (favored at low pH) (Fig. S3). The fraction of McCP-β in the 5cNO form (determined from the 395:416 absorbance ratio) was plotted as a function of pH and fitted to a simple single proton transition with a p*K*_a_ of 7.17 ± 0.03 (Fig. S3). The simplest explanation is that the p*K*_a_ corresponds to deprotonation of the unbound His 123 Nε in the 5cNO state, which allows His coordination to Fe in the 6cNO state. Low-temperature (10 K) EPR spectra of the Fe(II)NO complex also indicate a pH-dependent 6cNO/5cNO equilibrium, with a typical 5cNO three-line hyperfine pattern seen at pH 4 to 6, shifting to a line shape associated with 6cNO geometry, although with an unresolved nine-line hyperfine pattern, at pH 8 to 10 (Fig. S4) (28, 29).

**Figure 4:**
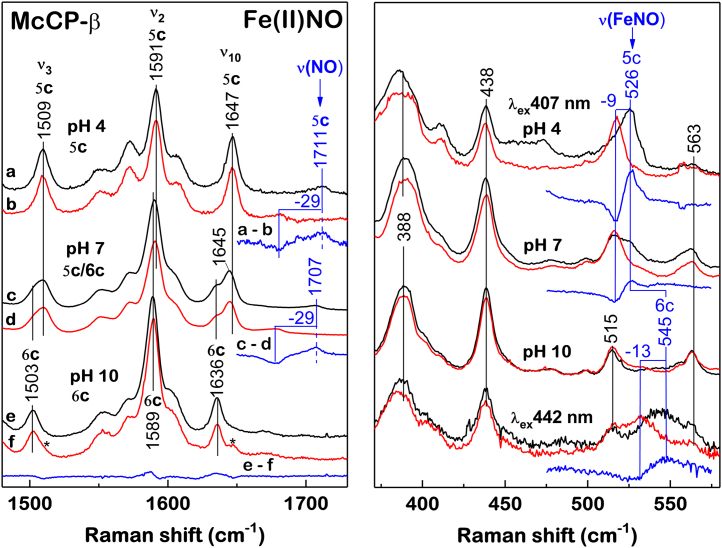
**Room-temperature resonance Raman spectra of Fe(II)NO McCP-β prepared with**^**14**^**NO (*black*) and**^**15**^**NO (*red*), together with**^**14**^**NO—**^**15**^**NO difference spectra (*blue*) in the high frequency (*left panel*) and low frequency (*right panel*) regions.** Spectra at pH 4.0 correspond to a 5cNO complex, whereas spectra at pH 10 correspond to a 6cNO complex (*asterisks* denote minor contributions of 5cNO species). A mixture of 5cNO and 6cNO species is observed at pH 7.0. All RR spectra were recorded with 407 nm excitation, except for the low-frequency region of the Fe(II)NO complex at pH 10, which was recorded using both 407 and 442 nm, the latter revealing a 6cNO ν(FeNO) mode at 545 cm^−1^. McCP-β, cyt *c*′-β from *Methylococcus capsulatus* (Bath).

RR measurements obtained with 407 nm laser excitation (Fig. 4) reveal additional structural information on the 6cNO and 5cNO complexes in solution. At pH 4.0, porphyrin marker bands are typical of a 5cNO complex: ν_3_ (1509 cm^−1^), ν_2_ (1591 cm^−1^), and ν_10_ (1647 cm^−1^) (Figs. 4 and S2), whereas at pH 10.0, porphyrin marker bands consistent with a 6cNO species are observed: ν_3_ (1503 cm^−1^) and ν_10_ (1636 cm^−1^) (Figs. 4 and S2). RR spectra obtained at pH 7.0 (close to the calculated equilibrium p*K*_a_) are consistent with the presence of both 5cNO and 6cNO species (Figs. 4 and S2). It is noted that the RR spectrum of 5cNO McCP-β at pH 4.0 shows a split ν_4_ mode (1360/1375 cm^−1^) (Fig. S2). The 1360 cm^−1^ component is distinct from the previously reported ν_4_ mode of 5c Fe(II) McCP-β (1353 cm^−1^) (4) and is instead reminiscent of the ν_4_ mode of four-coordinate heme (Fig. S2) (30). This suggests that the 5cNO RR sample undergoes heme–NO photodissociation, and that both the NO and proximal His ligand remain unbound in a significant fraction of the heme sites while the sample remains in the laser beam. Given that no laser-induced photochemistry was observed in RR spectra of the Fe(II)NO complex at pH 7.0, the increased build-up of the four-coordinate heme photoproduct at pH 4.0 might stem from protonation of the His ligand (thereby inhibiting His rebinding).

Vibrations of the FeNO moieties of the 5cNO and 6cNO complexes were identified *via* isotopic replacement with ^15^NO (Fig. 4). At pH 10.0 (6cNO population predominant), substitution with ^15^NO results in a 13 cm^−1^ downshift in an RR mode at 545 cm^−1^ (Fig. 4). This RR band is observed with both 407 and 442 nm laser excitation and has a wavenumber typical of a mixed bending/stretching ν(FeNO) vibration of a 6cNO complex. We did not observe any RR bands characteristic of an ν(NO) mode from the 6cNO McCP-β population. The ν(NO) RR modes of 6cNO heme proteins typically occur in the ∼1600 to 1630 cm^−1^ region, although they are known to be only weakly resonance enhanced (22). At pH 4.0 (5cNO population predominant), substitution with ^15^NO identifies a single 5cNO ν(NO) mode at 1711 cm^−1^ (with a 29 cm^−1^ downshift) and a single ν(FeNO) mode at ∼526 cm^−1^ (with a 9 cm^−1^ downshift) (Fig. 4). The 5cNO population present at pH 7.0 also exhibits ν(NO) and ν(FeNO) modes at similar wavenumbers to those observed at pH 4.0 (Fig. 4).

Analogous to the vibrational properties of 6cCO complexes, an inverse correlation between ν(NO) and ν(FeNO) frequencies has been demonstrated for model porphyrin 5cNO complexes with different Fe(II)→XO(π∗) backbonding strengths (21, 31). Although the relationship between ν(NO) and ν(FeNO) frequencies appears to be more complex in 5cNO heme protein environments (32), possibly because of variations in Fe–N–O angle and NO(π∗)→Fe(II)(d_z_^2^) σ bonding (33), the unusually high ν(NO) frequency (1711 cm^−1^) of the 5cNO McCP-β complex in solution is consistent with diminished Fe(II)→XO(π∗) backbonding (32), mirroring the trend observed for one of the ν(CO) modes of the 6cCO complex. Significantly, this suggests that the 5cNO McCP-β complex has NO bound on the distal face, and that the NO ligand interacts with the Phe 32 aromatic quadrupole in both subunits of the homodimer (in a similar manner to the NO ligand in the 6cNO crystal structure). Despite extensive efforts, we were unable to determine the structure of the 5cNO McCP-β species at 100 K with crystals grown at pH 6.5, even though this pH favors the 5cNO population in solution. RR spectra obtained at pH 7.0 show no evidence for change in the 6cNO:5cNO ratio upon cooling from room temperature to 100 K (data not shown). Instead, it is likely that the constraints of the crystalline lattice prevent dissociation of the proximal His 123 ligand. Nevertheless, our RR measurements of the 5cNO species indicate that the NO ligand is retained on the distal face of McCP-β, in contrast to cyt *c*′-α where NO switches to the proximal side.

### Kinetic parameters and *K*_*d*_ values for NO, CO, and O_2_ complexes

**Figure 5:**
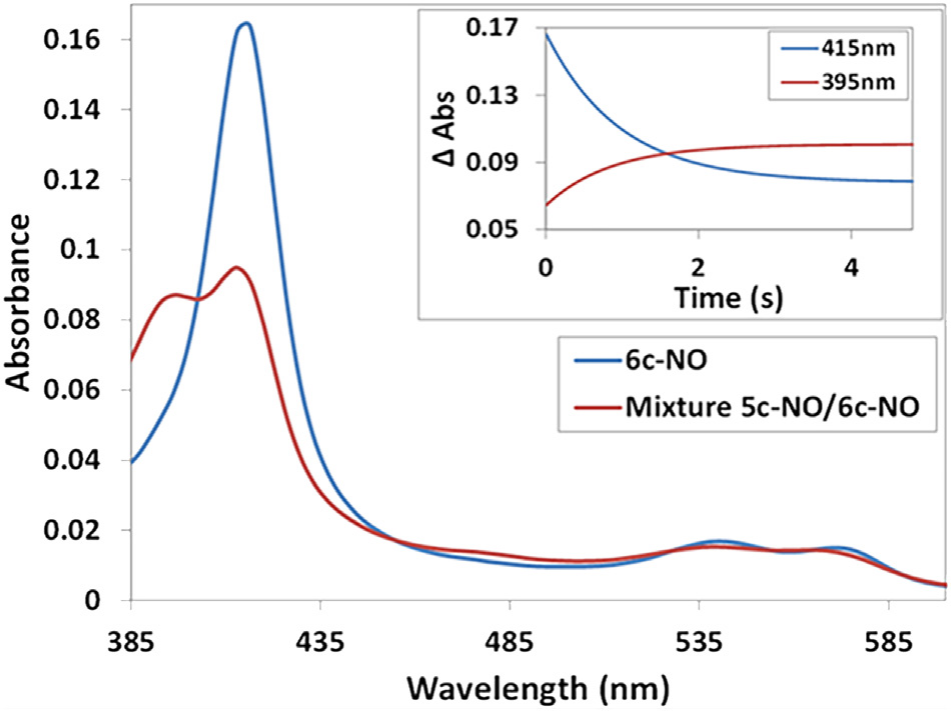
**Stopped-flow spectroscopy data for the reaction of ferrous McCP-β with NO at pH 7.5 showing the spectra obtained from global fitting of diode array data to a simple a → b model where a represents the initial 6cNO complex and b the final spectrum at the end of the reaction.** The *inset* shows representative time courses collected at 415 and 395 nm, together with fits to single exponential functions. The observed rate constant for 6cNO→5cNO conversion, measured at pH 7.5, remains effectively unchanged (*k*_obs_ ∼0.6 ± 0.05 s^−1^) when the NO concentration is varied from 0.01 to 0.05 mM. McCP-β, cyt *c*′-β from *Methylococcus capsulatus* (Bath).

The kinetics of gas binding to Fe(II) McCP-β were investigated using stopped-flow UV–visible absorbance measurements. In all cases, on rates were unusually high, consistent with relatively few steric constraints around the distal pocket. Stopped-flow measurements of NO binding to Fe(II) McCP-β showed that the 6cNO adduct had completely formed in the initial spectrum captured after mixing (Fig. 5). This was true at all pH values between 5 and 9 and for the lowest NO concentration employed (5 μM). Thus, the initial binding of NO to the vacant distal coordination site is extremely rapid, occurring within the instrument dead time (∼1.5 ms), which implies a second-order rate constant, *k*_on_(NO) ≥1 × 10^8^ M^−1^ s^−1^ (Table 4). Following the extremely rapid 6cNO formation, slower single exponential 6cNO→5cNO conversion (Fig. 5) establishes the pH-dependent 6cNO–5cNO equilibrium observed in static UV–visible spectra (Fig. S3). The observed rate constant for 6cNO→5cNO conversion, measured at pH 7.5, remains effectively unchanged (*k*_obs_ ∼0.65 ± 0.05 s^−1^) when the NO concentration is varied from 0.01 to 0.05 mM (0.01 mM = 0.7 s^−1^, 0.02 mM = 0.66 s^−1^, and 0.05 mM = 0.6 s^−1^), consistent with this being the first-order rate constant for histidine dissociation from the heme. Consequently, kinetic data (along with RR measurements) support a distal location for the 5cNO complex. Kinetics of NO release were determined by ligand replacement with excess CO (1.0 mM) in the presence of excess sodium dithionite as NO scavenger. At pH 9.5 (where only the 6cNO complex is present), the rate of NO release was monitored *via* the increase in 6cCO absorbance at 418 nm, with a single exponential fit of the 418 nm time course yielding a 6cNO *k*_off_ value of 0.011 ± 0.001 s^−1^ (Fig. S5, *top panel*). The rate constant value was independent of dithionite concentration (5–20 mM), consistent with NO release from the 6cNO complex as the rate-determining step. By contrast, at pH 5.0 (where only the 5cNO complex is present), Fe(II)NO→Fe(II)CO conversion is significantly slower than at pH 9.5. In this case, observed rate constants, *k*_obs_, increase with dithionite concentration: 0.00016 s^−1^ (5 mM dithionite), 0.00023 s^−1^ (14.5 mM dithionite), and 0.00042 s^−1^ (58 mM dithionite). This behavior indicates that dithionite reacts directly with the 5cNO species, such that NO release is not the rate-determining step.

**Table 4:** Kinetic parameters for six-coordinate Fe(II)CO and Fe(II)NO complexes of McCP-β and selected heme proteins

Protein	6cCO			6cNO			
	*k*_on_(CO) (M^−1^ s^−1^)	*k*_off_(CO) (s^−1^)	*K*_*d*_(CO) (M)	*k*_on_(NO) (M^−1^ s^−1^)	*k*_off_(NO) (s^−1^)	*K*_*d*_(NO) (M)	References
McCP-β (wt)	≥2.5 × 10^7^	0.20 ± 0.01	≤8 × 10^-9^	≥1 × 10^8^	0.011 ± 0.001	≤1 × 10^−10^	tw
F61V		0.32 ± 0.01			0.016 ± 0.001		tw
F32V		0.13 ± 0.01			0.0045 ± 0.0001		tw
AxCP-α (wt)	101	0.028	2.8 × 10^−4^	4.3 × 10^4^	0.0060	1.4 × 10^−6^	(9, 56)
L16A	1.1 × 10^6^	3.7 × 10^−6^	3.4 × 10^−12^	2.9 × 10^6^	2 × 10^−7^	7 × 10^−14^	(9, 10)
Mb pig (wt)	7.8 × 10^5^	0.019	2.4 × 10^−8^	1.7 × 10^7^			(57)
V68T	6.1 × 10^5^	0.079	1.3 × 10^−7^	4.9 × 10^6^			(57)
H64V, V68T	2.7 × 10^7^	0.063	2.3 × 10^−9^				(25)
sGC	4 × 10^4^	10.7	2.6 × 10^−4^	4.8 × 10^8^	27	5.6 × 10^−8^	(17, 58)
*Ns* H-NOX	3 × 10^6^	3.6	1.4 × 10^−6^	3 × 10^8^	0.05	1.7 × 10^−10^	(16)

Similar to the rapid nature of NO binding to McCP-β, stopped-flow measurements of CO binding show that the process was mostly complete within the dead time of the apparatus even at the lowest CO concentration employed (20 μM), indicating that the second-order rate constant for 6cCO formation, *k*_on_(CO), is ≥2.5 × 10^7^ M^−1^ s^−1^ (Table 4). The off-rate constant (*k*_off_) for the CO complex of wt McCP-β was determined at pH 7.0 by ligand replacement with excess NO. The presence of NO results in conversion of the Fe(II)CO complex (λ_max_ = 418 nm) to the Fe(II)NO state, which at pH 7.0 exists as a mixture of 6cNO (λ_max_ ∼416 nm) and 5cNO (λ_max_ ∼395 nm) populations (Fig. S5, *bottom panel*). Fe(II)CO→Fe(II)NO conversion was monitored *via* the increase in absorption at 385 nm, with a single exponential fit of the 385 nm time course yielding a *k*_off_ value of 0.20 ± 0.01 s^−1^ (Fig. S5, *bottom panel*). The rate constant was insensitive to variations in NO concentration (0.45–0.90 mM), consistent with CO release from the Fe(II)CO complex as the rate-determining step. Although the wt Fe(II)CO complex contains two populations with different Fe–CO bond strengths and Phe 32 orientations *(vide supra*), the observation of only monophasic (not biphasic) kinetics suggests that the two populations are in rapid equilibrium, and/or that any difference in *k*_off_(CO) values resulting from the Phe 32 orientation is too small to be resolved from our measurements.

**Figure 6:**
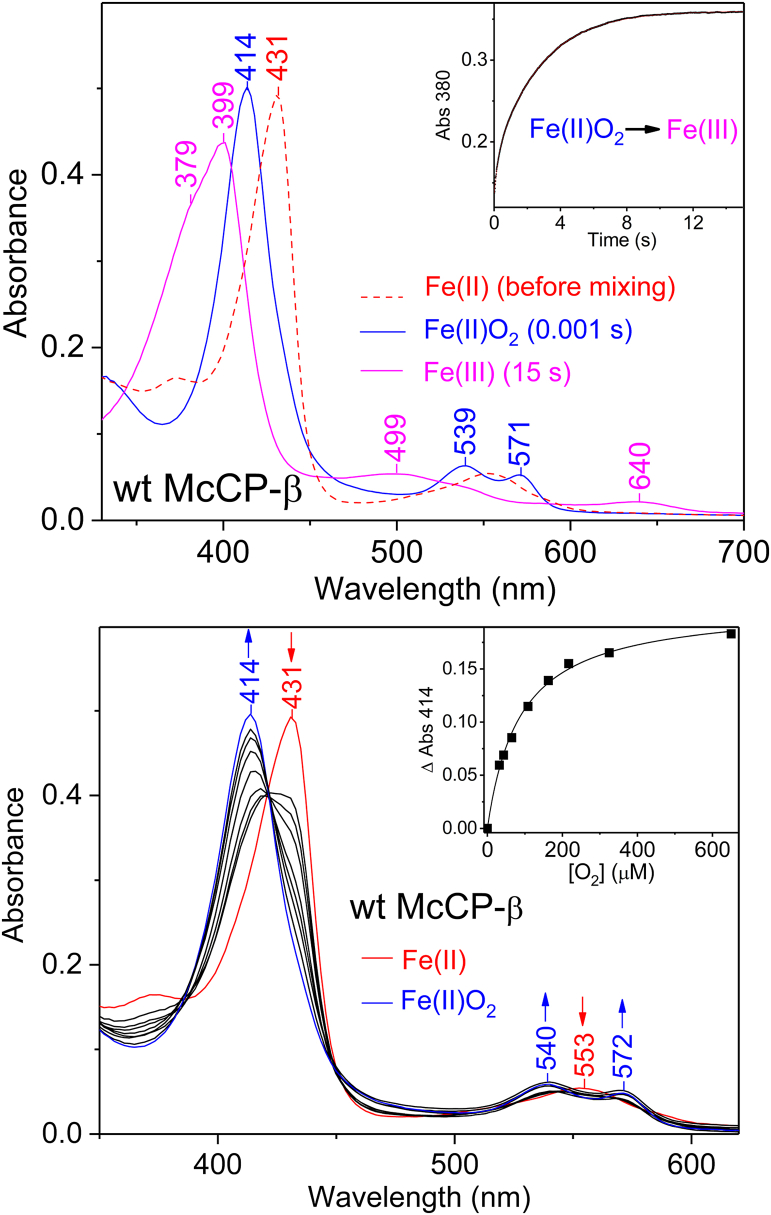
**Stopped-flow UV–visible characterization of the transient Fe(II)O**_**2**_**complex of wt McCP-β (25 °C, pH 8.9).***Top panel,* upon reaction with 650 μM O_2_, the Fe(II) state (*red dashed trace*) converts to an Fe(II)O_2_ complex (*blue trace*) within the stopped-flow mixing time, followed by biphasic autoxidation to the Fe(III) state (*magenta trace*) with rate constants, *k*_ox_(1) = 4.8 s^−1^ (20% ΔAbs) and *k*_ox_(2) = 0.37 s^−1^ (80% ΔAbs). *Bottom panel,* UV–visible spectra of Fe(II) protein (*red*) and the initial species formed (1 ms after mixing) with various final O_2_ concentrations (32–650 μM). The *inset* shows an O_2_ binding saturation curve with the absorption increase at 414 nm plotted *versus* O_2_ concentration and fit to a hyperbolic function, ΔA_414_ = (ΔA_max_ × [O_2_])/*K*_*d*_ + [O_2_] (*solid line*). An O_2_ binding curve was also prepared using the change in absorption at 430 nm (data not shown). The average *K*_*d*_ value obtained from these binding curves is 74 ± 13 μM. McCP-β, cyt *c*′-β from *Methylococcus capsulatus* (Bath).

Although heme–O_2_ coordination has not been reported for any cyt *c*′-β to date, a short-lived 6c Fe(II)O_2_ (6cO_2_) heme complex was observed using stopped-flow rapid mixing. Indeed, when Fe(II) McCP-β is reacted with 650 μM O_2_ at pH 8.9, the initial UV–visible spectrum (1 ms after mixing) exhibits absorbance features characteristic of a 6cO_2_ complex (λ_max_ = 414, 539, and 571 nm) (Fig. 6, *top panel*) and signifying that equilibration with O_2_ is complete within the stopped-flow mixing time. Subsequent UV–visible spectra show that the 6cO_2_ complex undergoes biphasic autoxidation to the Fe(III) state (λ_max_ = 399 nm) within 15 s, with rate constants, *k*_ox_(1) = 4.8 s^−1^ (20% ΔAbs) and *k*_ox_(2) = 0.37 s^−1^ (80% ΔAbs). The *K*_*d*_ value of the transient 6cO_2_ complex was determined by performing stopped-flow measurements using a range of nonsaturating O_2_ concentrations (32–650 μM). UV–visible spectra recorded 1 ms after mixing Fe(II) protein and O_2_ were used to determine the *K*_*d*_ value from an O_2_-binding saturation curve by plotting the absorbance increase at 414 nm, Δ_414_ (relative to unbound Fe(II) protein) as a function of [O_2_] (Fig. 6, *bottom panel*), and fitting to a hyperbolic function (Equation 1)

(1) $${\Delta A}_{414}=\frac{{\Delta A}_{\max}\times \left[O_{2}\right]}{K_{d}+\left[O_{2}\right]}$$

An O_2_-binding curve was also prepared using the change in absorption at 430 nm (data not shown). The average *K*_*d*_ value obtained from these binding curves is 7.4 (±1.3) × 10^−5^ M. Since *K*_*d*_ values for heme–gas complexes can be calculated from the *k*_off_/*k*_on_ ratio, our experimentally determined *k*_off_ values for the 6cNO and 6cCO complexes, together with the lower limit estimates of *k*_on_, yield upper limits of *K*_*d*_ for the 6cCO (≤8 × 10^−9^ M) and 6cNO (≤1 × 10^−10^ M) species (Table 4). Previous studies of heme systems have identified a “sliding scale” relationship between the reactivities of O_2_, CO, and NO that can be used to predict *k*_on_, *k*_off_, and *K*_*d*_ values for different heme pocket environments (13, 17, 34). In order to model reactivity trends in McCP-β, we used reported data for the L16A variant of AxCP-α (Table 4), which like McCP-β contains a sterically accessible distal heme pocket devoid of H-bond donors (9, 10). Using our experimentally determined *K*_*d*_ value for the McCP-β 6cO_2_ complex (7.4 × 10^−5^ M) (Table 4), together with the ratio of *K*_*d*_ values (O_2_:CO:NO) reported for L16A AxCP-α (70,000:49:1), yields predicted *K*_*d*_ values for the 6cNO complex (∼1 × 10^−10^ M) and the 6cCO complex (∼5 × 10^−^^9^ M), both of which agree with the limiting estimates from our stopped-flow measurements. The sliding scale rule was also used to predict *k*_on_ and *k*_off_ values for the transient 6cO_2_ McCP-β complex. In line with the ratio of L16A *k*_off_ values (O_2_:CO:NO) of 85,000:19:1, our experimentally determined McCP-β *k*_off_ values for the 6cCO complex (0.20 s^−1^) and the 6cNO complex (0.011 s^−1^) exhibit an 18:1 ratio (similar to that of L16A), while predicting a *k*_off_ value of ∼9000 s^−1^ for the 6cO_2_ McCP-β complex. The fact that the predicted McCP-β O_2_ off rate is much higher than that of NO and CO reflects the inherently weaker Fe(II)→XO(π∗) backbonding of O_2_ complexes (because of a doubly occupied π∗ orbital) and the lack of H-bond stabilization of the polar Fe(II)O_2_ unit within the McCP-β distal pocket (35).

### Effect of F32V and F61V mutations on McCP-β structure and reactivity

We further probed the roles of the distal Phe cap residues (Phe 61 and Phe 32) by comparing the properties of wt McCP-β with those of the F32V and F61V variants, focusing on NO and CO complexes. Our goal in characterizing these aromatic → aliphatic variants was twofold: (i) to provide further evidence that the Phe 32 aromatic quadrupole limits Fe(II)→XO(π∗) backbonding and (ii) to establish the extent to which the Phe 32 aromatic quadrupole influences heme–gas reactivity.

#### F32V and F61V variants in the absence of gas ligands

Compared with wt McCP-β, both mutations maintain a very similar overall fold in the ligand-free chemically reduced form, with structural changes essentially limited to the distal heme pocket. These result in a less crowded distal pocket and better accessibility to the heme from the surrounding solvent, although no water is evident within the Fe(II) heme pockets (Fig. S6). The as-isolated Fe(III) forms of the F61V and F32V variants also have spectroscopic features similar to those previously reported for the wt protein, with RR spectra consistent with 5cHS Fe(III) heme (with no distal solvent ligand) and EPR spectra showing a mixture of two HS species with different rhombicities (Fig. S7). In the resting state of the F61V variant, there is a change in the positioning of Phe 32; this is now able to take up two possible conformations, distinct from that of the wt McCP-β structure. In heme A of the ligand-free chemically reduced state, one is moved outward from the heme by 1.2 Å and rotated by 80° around the Cβ–Cγ bond from the native structure position whilst the other has moved into the space above the heme created by the Val mutation, with the face of the ring rotating by 103° compared with the native structure (Fig. S6). In heme B, one has rotated outward from the heme by 82°, whereas the other is again sitting over the face of the heme, where Phe61 would previously have been, having rotated by 75°. The F32V mutation causes no other changes to residues around the heme with the Val sitting in the same relative position as the Phe in the native structure in the ligand-free chemically reduced form (Fig. S6).

#### Fe(II)CO complexes of F32V and F61V variants

**Figure 7:**
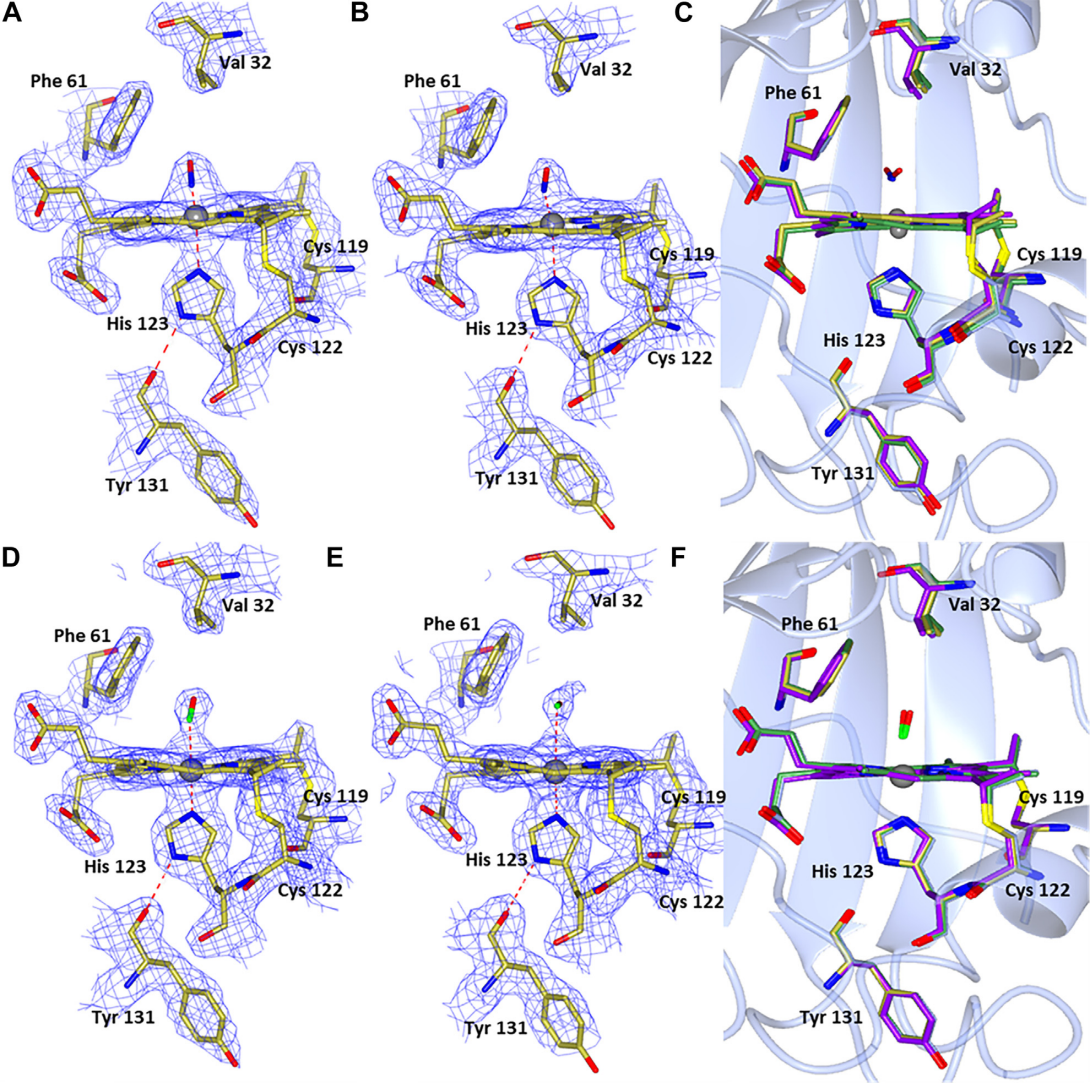
**Hemes A and B of F32V McCP-β.** With either NO (*A* and *B*) or CO bound (*D* and *E*). Comparison of Fe(II) F32V hemes A (*blue*) and B (*green*) and both hemes A (*purple*) and B (*gold*) with NO (*C*) or CO (*F*) bound. Only one orientation of CO can be seen with a slightly more bent geometry than native McCP-β at angles of 164/165°. Two orientations of NO can be seen: one points toward Leu 28 (heme A) and the other toward Gly 82 (heme B). These have Fe–N distances of 1.90 and 1.97 Å and Fe–N–O angles of 110° and 92° in hemes A and B, respectively. McCP-β, cyt *c*′-β from *Methylococcus capsulatus* (Bath).

**Figure 8:**
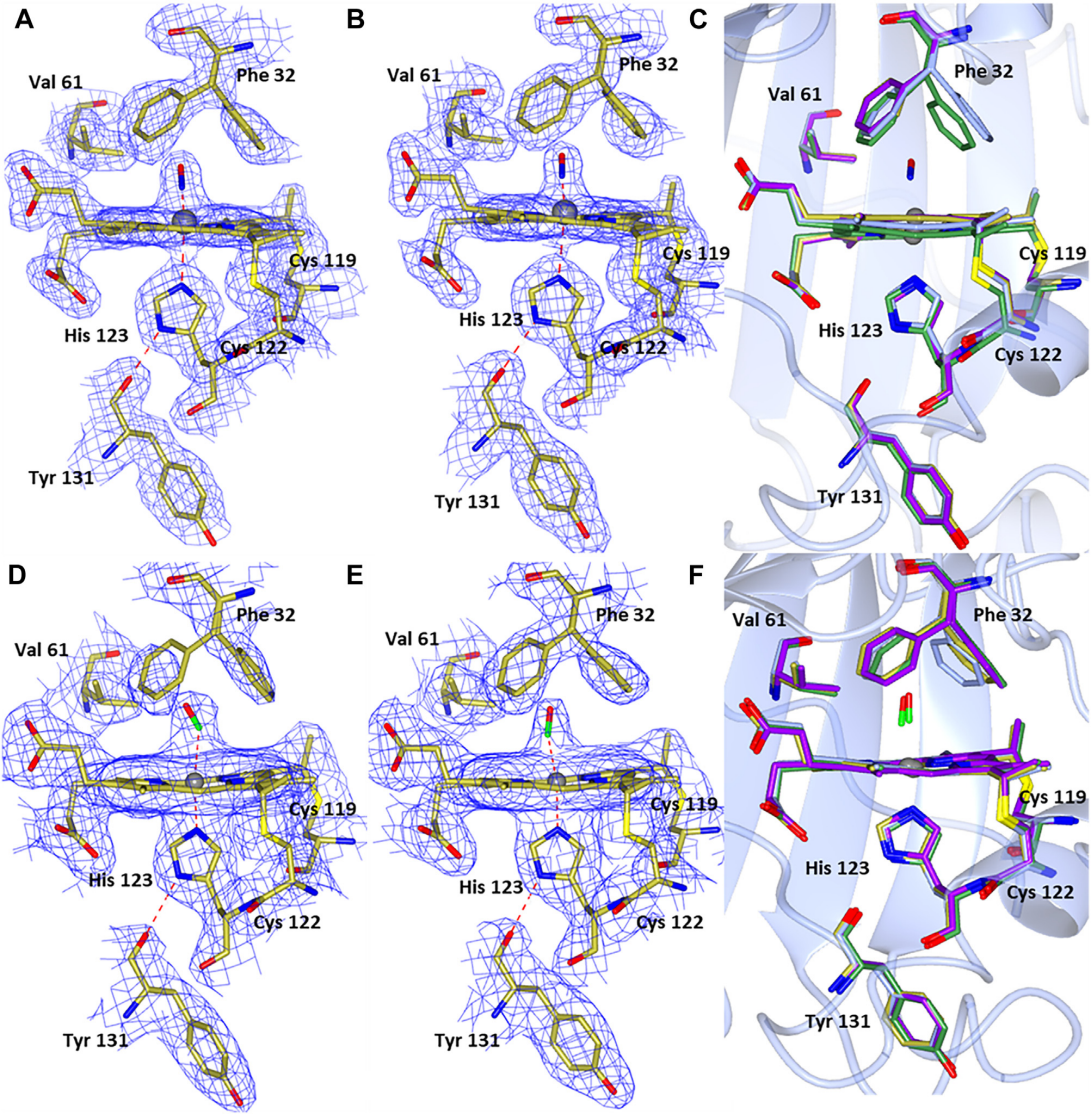
**Hemes A and B of F61V McCP-β**. With either NO (*A* and *B*) or CO bound (*D* and *E*). Comparison of Fe(II) F61V hemes A (*blue*) and B (*green*) and both hemes A (*purple*) and B (*gold*) with NO (*C*) or CO (*F*) bound. Only one orientation of both NO and CO can be seen with angles of 166° and 177° (CO) and 130° and 135° (NO). McCP-β, cyt *c*′-β from *Methylococcus capsulatus* (Bath).

In the crystal structure of the F32V variant 6cCO complex (Fig. 7), the homodimer exhibits Fe–C distances of 2.03 and 2.07 Å, and a slightly more bent geometry than the native ligand–bound structure with Fe–C–O angles of 164° and 165° for monomer A and B, respectively. There is no observable movement of the pocket residues upon ligand binding, and only one orientation of Phe 61 is observed. The F61V variant exhibits Fe–C distances of 2.01 and 2.00 Å and a similar bent geometry to the native ligand–bound structure with Fe–C–O angles of 166° and 177° for monomer A and B, respectively (Fig. 8). The F61V variant still displays two possible conformations of Phe 32 in the presence of CO. In heme A, one is in a similar position to the native CO structure, although the plane of the Phe ring is facing more toward the heme with the nearest ring atom being at a distance of 3.43 Å, whereas the one which had moved into the space above the heme (previously occupied by Phe 61) has also rotated so as to present the plane of the Phe ring to the CO oxygen with the nearest ring atom being 3.23 Å away. In heme B, there are again two conformations of Phe 32 in similar positions; again one is in a similar position to the native CO structure, with just a slight rotation so that the plane of the Phe ring is presenting less to the CO oxygen with the nearest ring atom being 3.14 Å away, whereas the other is in the space above the heme and slightly rotated so as to present more of the plane to the CO oxygen with the nearest ring atom being 3.08 Å away from the CO oxygen.

Both the F61V and F32V variants form Fe(II)CO complexes with absorption features similar to those of the wt complex. RR measurements of F61V and F32V Fe(II)CO complexes support our hypothesis that the local negative polarity of the Phe 32 aromatic quadrupole limits buildup of charge associated with Fe(II)→CO(π∗) backbonding, thereby weakening the Fe–CO bond. Fig. S8 shows the identification of the ν(FeCO) RR vibrations in wt, F61V, and F32V McCP-β using isotopic substitution with ^13^CO. In the case of the F61V variant, a doublet of ν(FeCO) frequencies is identified at 483 cm^−1^ and 495 cm^−1^, from isotope shifts of −4 cm^−1^ and 3 cm^−1^, respectively). The pair of ν(FeCO) frequencies observed for the F61V variant (483 and 495 cm^−1^) are similar to those of the ν(FeCO) doublet for wt McCP-β (481 and 491 cm^−1^) (Table 3), with the lower frequency ascribed to CO interacting with the local negative polarity of the aromatic quadrupole of Phe 32.

Similar to the wt Fe(II)CO complex, two orientations of Phe 32 can be seen in the F61V crystal structure (Fig. 8). However, unlike the wt structure, where a unique Phe 32 orientation was seen in each monomer, F61V displays two orientations in both monomers. By contrast, the F32V variant exhibits only a single broad ν(FeCO) RR band at 497 cm^−1^ with an isotope shift of −4 cm^−1^ (Fig. S4). This corresponds to the single orientation of the remaining Phe residue seen in the F32V CO complex structure. The 497 cm^−1^ frequency is characteristic of CO bound within a neutral hydrophobic microenvironment (as can be seen in the F32V CO complex structure) and is consistent with the removal of the Phe 32 aromatic quadrupole *via* the F32V mutation. Attempts to identify ν(CO) vibrations in the 1950 to 2000 cm^−1^ region were unsuccessful because of poor signal-to-noise ratios. Along with the *k*_off_ value for the 6cCO complex of wt McCP-β (0.20 ± 0.01 s^−1^), we also determined the corresponding *k*_off_ values for the F61V (0.32 ± 0.01 s^−1^) and F32V (0.13 ± 0.01 s^−1^) variants (Fig. S9 and Table 4). In line with RR data that indicate a modest weakening of the Fe–CO bond (because of the Phe 32 aromatic quadrupole), the CO off rates of wt and F61V are 1.5 (±0.2)-fold and 2.5 (±0.3)-fold higher than that of F32V (Table 4).

#### Fe(II)NO complexes of F32V and F61V variants

NO is bound to the distal face of the heme in both the F61V and F32V variants. In F61V, only one conformation of NO binding can be seen in each heme, pointing toward Gly 82 (Fig. 8). These both have Fe–N distances of 1.86 Å and Fe–N–O angles of 130° and 135° in hemes A and B, respectively. Only one conformation of the remaining Phe can be seen in each heme coming across the face of the heme toward the mutated Val residue. In F32V, only one conformation of NO binding can again be seen in each heme. However, one points toward Leu 28 (heme A) and the other toward Gly 82 (heme B) (Fig. 7). These have Fe–N distances of 1.90 and 1.97 Å and Fe–N–O angles of 110° and 92° in hemes A and B, respectively. There is no movement of the pocket residues observed in the F32V variant upon introduction of NO. In both NO-bound variants, the Fe–His distance appears longer than expected for a six-coordinate structure suggesting the His residue may be dissociating from the heme, giving rise to a five-coordinate form. This is most clearly evident in the F61V structure with Fe–His distances of 2.93 and 3.03 Å in hemes A and B, respectively. It can be seen in the structures that the heme becomes domed as the Fe moves upward (Fig. S10), resulting in the extended Fe–His distances. The His residue displays a slight rotation of ∼8° around the Cβ–Cγ bond and only moves downward by about 0.2 Å. There is also a small amount of movement in the rest of the chain around the His residue. Notably, the residues remain in a position to allow His 123 to remain hydrogen bonded to Tyr 131.

One feature that distinguishes the Fe(II)NO complexes of the F61V and F32V variants in solution is their pH-dependent properties. UV–visible absorption spectra indicate that the F61V variant exhibits a pH-dependent equilibrium between 6cNO and 5cNO species, with a p*K*_a_ value (∼7) similar to that of wt protein (Fig. S11). By contrast, the F32V variant forms a predominantly 6cNO species at pH 7, with a p*K*_a_ of ∼6 for the 5cNO to 6cNO transition (Fig. S11). These differences are also apparent in low-temperature EPR spectra of the F61V and F32V Fe(II)NO complexes (Fig. S12). Because of instability of the F32V 5cNO species at low pH, we focused our comparison on the kinetic properties (*k*_off_ values) of the 6cNO species. The influence of Phe cap mutations on 6cNO *k*_off_ values exhibits a similar trend to that of the 6cCO *k*_off_ values (*vide supra*). Relative to the wt 6cNO *k*_off_ value (0.011 ± 0.001 s^−1^), the F61V variant exhibits a *k*_off_ value of 0.016 ± 0.001 s^−1^ and F32V variant a value of 0.0045 (±0.0001) s^−1^ (Fig. S13 and Table 4). Thus, relative to the F32V variant, the 6cNO Fe(II)NO *k*_off_ values are higher by factors of 2.4 (±0.3) (wt) and 3.6 (±0.3) (F61V). The trend toward slower NO release in the F32V variant is consistent with the predicted increase in Fe–NO bond strength following removal of the Phe 32 aromatic quadrupole.

## Discussion

### Influence of the McCP-β distal Phe cap on heme reactivity with diatomic gas ligands

McCP-β exhibits exceptionally high *k*_on_ values for diatomic gas binding that approach the diffusion limit (Table 4). The high on rates indicate that the distal Phe cap of McCP-β provides relatively little steric hindrance to heme–gas coordination. This contrasts with the significant steric constraints to gas binding imposed by distal pocket residues in α-helical cyts *c*′-α (7, 8, 9, 10, 11, 12). Within the McCP-β distal pocket, crystal structures of the 6cNO and 6cCO complexes reveal that the conformation of Phe 61 is essentially unchanged upon the coordination of NO or CO to heme, whereas Phe 32 undergoes a rotation around the Cβ–Cγ bond. Phe 32 remains in close proximity to the bound gas (within 3.5 Å of the ring face atoms), with its ring face oriented toward the NO ligand in both subunits of the homodimer, and toward CO in one of the subunits. Significantly, RR measurements of NO and CO complexes show evidence of unusually weak Fe(II)→XO(π∗) backbonding, which we attribute to proximity of the gas ligand to the local negative polarity of the aromatic quadrupole of the Phe 32 ring. Given the two Phe 32 orientations evident in the 6cCO structure, the observation of *two sets* of ν(CO) and ν(FeCO) RR bands further suggests that Phe 32 inhibits Fe(II)→XO(π∗) backbonding only when its ring face presents to the XO ligand. The impact of the local negative polarity of the Phe 32 aromatic quadrupole on heme–CO vibrations is similar to that of nonbonded electrons in the V68T variant of pig Mb (∼20 cm^−1^ upshift in ν(CO) and ∼10 cm^−1^ downshift in ν(FeCO)) (Table 3) (25, 26).

We assessed the impact of the Phe 32 aromatic quadrupole on McCP-β heme–gas affinity by comparing the structural, spectroscopic, and kinetic properties of wt McCP-β with those of the F61V and F32V variants. Crystallographic and RR data confirm that NO and CO ligands interact with the Phe 32 ring face in the F61V variant but are unaffected by any aromatic quadrupole in the F32V variant. Accordingly, for NO and CO ligands, we reasoned that diminished Fe(II)→XO(π∗) backbonding in wt and F61V (because of the negative polarity of the Phe 32 quadrupole) should elevate *k*_off_ values relative to F32V. Indeed, *k*_off_ values for the 6cCO and 6cNO complexes of wt and F61V McCP-β are consistently higher than those of the F32V variant, although the observed increases are quite modest, ranging from 1.5 (±0.2)-fold to 3.6 (±0.3)-fold (Table 4). We note that decreases in diatomic gas affinity of a similar magnitude are also associated with the introduction of negative polarity from nonbonded electrons in the V68T Mb variant. In line with weaker Fe(II)→CO(π∗) backbonding, *k*_off_(CO) values for the V68T (0.079 s^−1^) and H64V/V68T variant (0.063 s^−1^) increase by factors of ∼4 and ∼3, respectively, relative to wt Mb (0.019 s^−1^) (Table 4). Finally, we do not attribute the decrease in F32V off rates to steric effects. Although the replacement of the distal occluding Leu with smaller side chains leads to lower off rates in AxCP-α (Table 4) (9, 10, 12), this was attributed to trapping of the gas ligand caused by a change in propionate conformation (36)—an effect not observed in the F32V or F61V McCP-β structures.

### Determinants of 5cNO *versus* 6cNO coordination

Although 5cNO formation is the norm in model porphyrin Fe(II)NO complexes (because of the *trans* effect weakening of the bond opposite the NO), most heme proteins retain a 6cNO geometry because of conformational constraints of the protein scaffold that help retain the endogenous (His) protein ligand (37). In some heme proteins, such as Mb, 6cNO→5cNO conversion can be achieved by protonation of the proximal His ligand, although the p*K*_a_ for this conversion is relatively low (4.7 in the case of Mb) (37). By comparison, the 6cNO–5cNO equilibrium in McCP-β (attributed to protonation of the His 123 Nε) has an unusually high p*K*_a_ of ∼7.2. In the case of the Cys-ligated heme of nitric oxide synthase, a pH-dependent 6cNO→5cNO transition with an apparent p*K*_a_ above 7 was reported following a W409F mutation to remove a H-bond to the adjacent proximal Cys ligand, an effect attributed to weakening of the Fe(II)–Cys linkage (38). However, there is no evidence that 5cNO formation in McCP-β is due to an inherently weak proximal Fe(II)–His bond, since previous RR studies of McCP-β reveal a relatively high ν(Fe–His) frequency of 219 cm^−1^ (4). Analysis of the 6cNO McCP-β crystal structure does not indicate any obvious structural feature near the proximal heme face that might stabilize the unbound (protonated) state of the His ligand in the 5cNO population. The present study also indicates that McCP-β retains NO on the *distal* heme face, thereby ruling out a distal → proximal switch in heme–NO coordination as a driver of 5cNO formation, as occurs in cyts *c*′-α (12). It is possible that the relatively high p*K*_a_ for the 6cNO–5cNO equilibrium in McCP-β could arise from increased flexibility in the surrounding protein matrix. Interestingly, the F32V (but not the F61V) McCP-β mutation lowers the *p*K_a_ for the 6cNO–5cNO equilibrium from ∼7.0 (wt and F61V) to ∼6.0 (F32V). It is conceivable that the microenvironment of the Phe 32 quadrupole could interact differently with the Fe–NO units of 6cNO and 5cNO species to influence their relative stability and favor the 5cNO form. Future studies will investigate whether this effect is connected to the aromatic quadrupole or else to change in the stability of the surrounding protein. Factors controlling heme–NO coordination in McCP-β may be relevant to its possible physiological relevance during nitrification. Klotz *et al.* (3) showed using quantitative PCR that both *hao* (encoding hydroxylamine oxidoreductase) and *cytS* (encoding McCP-β) genes exhibited greatly increased transcript numbers when cells were exposed to ammonia, whereas in contrast, there was no increase for *cytL* encoding cytochrome P460. This may suggest a role of McCP-β in buffering NO (produced by hydroxylamine oxidoreductase during nitrification) or a response to other sources of nitrosative stress under these conditions.

### Influence of aromatic quadrupoles in heme-based gas sensors

Our characterization of McCP-β prompted us to search for evidence of aromatic quadrupole interactions in structural and spectroscopic data reported for other gas-binding heme proteins. We note that the distal pockets of the mammalian heme-based NO sensor, sGC, as well as the prokaryotic analog, *Ns* H-NOX, contain aromatic side chains in positions that could interact with diatomic gas ligands (14, 15). Crystal structures of *Ns* H-NOX show that the aromatic ring face of Trp 74 is presented toward heme-bound CO and NO (14). Although the precise location and conformation of gas ligands in sGC has yet to be resolved, recent cryo-electron microscopy of sGC indicates that the ring face of Phe 74 is in a position to interact with distally bound gases (15). In line with the predicted impact of aromatic quadrupoles in these proteins, RR data are indicative of diminished Fe(II)→XO(π∗) backbonding. In particular, unusually high ν(CO) frequencies, coupled with low ν(FeCO) frequencies, are observed for 6cCO complexes of sGC (1985/473 cm^−1^) and *Ns* H-NOX (1986/470 cm^−1^) (Table 3, and references therein), resembling the impact of the Phe 32 aromatic quadrupole in McCP-β. In addition, sGC (which forms a stable 5cNO complex) exhibits an unusually high ν(NO) frequency of 1700 cm^−1^ in the presence of GTP (39) also mirroring the behavior of McCP-β. Other members of the H-NOX family also exhibit unusually high ν(CO) frequencies but without an accompanying decrease in ν(FeCO). For example, the 6cCO complex of *Tt* H-NOX exhibits a ν(CO) frequency of 1989 cm^−1^ (similar to that of sGC and *Ns* H-NOX) but a substantially higher ν(FeCO) frequency of 490 cm^−1^ (∼20 cm^−1^ higher than that of sGC and *Ns* H-NOX) (40). In this case, mutagenesis studies of *Tt* H-NOX suggest that the high ν(CO) frequency is not because of heme pocket polarity but rather to strong H-bonding interactions between the heme propionates and the conserved YxSxR motif (41).

In summary, sGC and *Ns* H-NOX are the only structurally characterized members of the H-NOX family to date that show a negatively polarized aromatic ring face positioned to interact with gas ligands on the distal heme face. The vibrational properties of their CO and NO complexes are similar to those of McCP-β and are consistent with diminished Fe(II)→XO(π∗) backbonding resulting from the negatively polarized face of an aromatic quadrupole. We note that sGC and *Ns* H-NOX are both optimized to sense NO (rather than O_2_ or CO) (13, 17). The means by which sGC selectively binds and is activated by NO has attracted considerable interest. Significantly, NO activation of sGC requires Fe–His bond scission, which occurs when its transient 6cNO complex converts to a 5cNO species. This process also selectively boosts heme–NO affinity by virtue of the fact that 5cNO sGC complexes exhibit much lower *k*_off_ values (≤0.12 s^−1^) than the 6cNO precursor (27 s^−1^) (17, 42, 43). Interestingly, spectroscopic and kinetic measurements suggest that sGC can form 5cNO complexes on either heme face, with a proximal 5cNO species generated with excess NO (44), and a distal 5cNO species with stoichiometric NO (42) (a condition more akin to the physiological environment) (45). Furthermore, the distal 5cNO species has a much higher *k*_off_ value (0.12 s^−1^) than that of the proximal 5cNO species (6 × 10^−4^ s^−1^) (42, 43). Although the structural reasons for the high distal 5cNO *k*_off_ value remain to be determined, our present study raises the possibility that interaction of the distal NO ligand with the local negative polarity of the aromatic quadrupole of Phe74 might contribute in part to the relatively rapid NO release, which may enable sGC activation to be reversible under physiological conditions. Future mutagenesis studies of the Phe 74 residue in sGC, and the Trp 74 residue in *Ns* H-NOX, should investigate the specific influence of aromatic quadrupoles on heme–gas complexes in these two H-NOX proteins.

## Conclusions

A major determinant of heme protein reactivity with diatomic gases is the microenvironment of the distal heme pocket. In this study of the gas-binding heme protein, McCP-β, we probed the influence of a pair of aromatic residues (Phe 32 and Phe 61) that represent the so-called “distal Phe cap” found in many cyts *c*′-β. Stopped-flow measurements indicate that the binding of NO, CO, and O_2_ to Fe(II) McCP-β approaches the diffusion limit, consistent with relatively little steric hindrance to distal heme coordination. Whereas the Fe(II)O_2_ complex was not structurally characterized, because of its rapid autoxidation to the Fe(III) state, high-resolution crystal structures of the 6cNO and 6cCO complexes show that Phe 32 rotates to present its aromatic ring face toward the gas ligand. RR data for the 6cCO and 5cNO complexes in solution (the latter favored at pH <7) reveal unusually weak Fe(II)→XO(π∗) backbonding, which we attribute to the local negative polarity of the Phe 32 aromatic π-system. A comparison of the gas-binding properties of wt McCP-β with those of the F32V and F61V variants suggests that the Phe 32 aromatic quadrupole acts to lower the affinity for NO and CO by speeding up gas release. Although the *k*_off_ increases attributed to the Phe 32 aromatic quadrupole are quite modest (2.4–3.6 (±0.3)-fold for the 6cNO complex), an elevated NO off rate could be one of several factors regulating the putative NO-binding function of McCP-β (3). A role for Phe 32 in modulating the relative populations of 6cNO–5cNO species is also a possibility suggested by the present study. Interestingly, the distal heme pockets of the mammalian NO sensor, sGC and the bacterial analog, *Ns* H-NOX, also contain aromatic residues in positions to interact with diatomic gas ligands. Given that RR data reported for NO and CO complexes of sGC and *Ns* H-NOX are also consistent with unusually weak Fe(II)→XO(π∗) backbonding, we propose that heme–XO coordination in sGC and *Ns* H-NOX is also impacted by the local negative polarity of an aromatic quadrupole. Future mutagenesis studies of sGC and *Ns* H-NOX involving heme pocket aromatic → aliphatic substitutions should shed further light on the influence of aromatic quadrupoles in these proteins. Overall, our results identify a novel determinant of heme–XO reactivity in cyts *c*′-β (negatively polarized aromatic quadrupole), with possible implications for molecular recognition and activation of mammalian and bacterial NO sensors.

## Experimental procedures

### Protein expression and purification

Recombinant McCP-β was expressed, purified, and crystallized as described previously (4). Mutagenesis to produce the F32V and F61V variants was carried out using the QuickChange Lightning Kit (Agilent), and the presence of the desired mutation was confirmed through sequencing *via* Eurofins TubSeq service. Expression and purification of both variants was carried out as previously described for the wt protein (4). The F61V variant was crystallized under the same conditions as the wt protein as previously reported, whereas the F32V variant was crystallized under the following conditions: 0.01 M ZnSO_4_, 30% PEG 550 (v/v), and 0.1 M Mes, pH 6.5. In preparation for ligand soaking, crystals were transferred into glass vials containing degassed reservoir solution supplemented with 100 mM ascorbic acid, and the vials were sealed with a rubber seal. The crystals were then incubated at 18 °C for 2 h to reduce the protein crystals. To produce NO-bound McCP-β crystals, 100 μl of PROLI NONOate was added to one of the vials and incubated at 18 °C for 2 h. To produce CO-bound McCP-β crystals, CO gas was bubbled through another vial and was left to incubate for 2 h. Prior to X-ray data collection, crystals were cryoprotected by transfer to reservoir solution comprising mother liquor supplemented with 10% (v/v) glycerol and flash cooled in liquid nitrogen.

X-ray diffraction data for the native McCP-β were measured at Diamond Light Source beamline I02 using an X-ray wavelength of 0.9795 Å and a Pilatus 6M detector, and X-ray diffraction data for F32V and F61V variants were measured at Diamond Light Source beamlines I04 and I03 using an X-ray wavelength of 0.9795 Å and an Eiger2 XE 16M detector. Data were automatically processed in xia2 using DIALS, XDS, and Aimless (46, 47, 48).

All structures were refined by maximum likelihood methods using REFMAC5. Fe–His and Fe–XO restraints were relaxed during refinement (49). Model building between cycles of refinement, including addition of water molecules and ligands, was performed in Coot (https://www2.mrc-lmb.cam.ac.uk/personal/pemsley/coot/) (50), and the quality of the structures was monitored using the MOLPROBITY (51) and JCSG Quality Control servers. Coordinates and structure factors were deposited in the Protein Data Bank.

### pH dependence spectroscopy

A 5× cocktail buffer containing 500 mM CAPS, 250 mM Mes, 250 mM Hepes, 250 mM Pipes, and 250 mM TAPS was prepared to cover the desired pH ranges (MHTCP buffer). The cocktail buffer was diluted to 1× MHTCP and adjusted to pH 4, 5, 6, 7, 8, 9, and 10. Ferric protein was added to a final concentration of 5 μM in 1 ml of 1× MHTCP buffer. NO-bound samples were prepared by reducing the ferric protein sample by addition of sodium dithionite. About 10 μl of ∼80 mM PROLI NONOate was injected into the prereduced protein solution using a 10 μl Hamilton syringe to provide an excess of the ligand. 5× MHTCP cocktail buffer was diluted to 1× MHTCP and adjusted to pH 4, 5, 6 to 8 (at 0.1 pH unit intervals), 9, and 10. UV–visible absorption spectra were measured at each pH. To eliminate noise introduced into the dataset by repositioning the cuvette following each pH adjustment, the difference in absorbance between two wavelengths, on either side of the isosbestic point (415 and 395 nm), was used to construct the titration curve. A Varian Cary 50 spectrophotometer was used to measure UV–visible absorption spectra at each pH.

EPR spectra were measured on a Bruker EMX EPR spectrometer (X band) at 10 K. A spherical high-quality Bruker resonator ER 4122 SP 9703 and an Oxford Instruments liquid helium system were used to measure the low-temperature EPR spectra. Wilmad SQ EPR tubes (Wilmad Glass) were filled with the McCP-β solutions and frozen in methanol kept on dry ice. The tubes were then transferred to liquid nitrogen. The spectra were measured at the following instrumental conditions: microwave frequency ν_MW_ = 9.4668 GHz; microwave power P_MW_ = 3.17 mW; modulation frequency ν_M_ = 100 kHz; modulation amplitude A_M_ = 5 G; scan rate v = 22.6 G/s; time constant τ = 81.92 ms; conversion time, at a 2048 data point scan range, *t*_conv_ = 81.92 ms.

### RR spectroscopy

Protein samples for RR measurements (∼150 μM in heme) were prepared in solutions containing 0.10 M NaCl and buffered with either 50 mM acetate (pH 4.0), 50 mM Mops (pH 7.0), or 50 mM Ches (pH 10). Ferric McCP-β was reduced to the ferrous state inside an anaerobic glove box by reacting a concentrated protein stock solution (∼3 mM in heme) with a fivefold excess of sodium dithionite. After diluting the reduced protein to 150 μM with buffer solution and transferring to an anaerobic septum-sealed capillary tube, Fe(II)CO or Fe(II)NO complexes were generated by the introduction of gas (either ^12^CO, ^13^CO, ^14^NO, or ^15^NO). The identity of samples was confirmed by UV–visible spectroscopy before and after RR measurements using a modified Cary 50 spectrophotometer. RR spectra were recorded at room temperature in a 90° scattering geometry using a custom McPherson 2061/207 spectrograph (100 μM slit width, 0.67 m focal length, and 2400 grooves/mm grating) equipped with a Princeton Instruments liquid N_2_-cooled (LN1100PB) CCD detector. Excitation wavelengths were provided by krypton ion (406.7 nm) and He–Cd (441.6 nm) lasers. RR spectra of Fe(II)CO and Fe(II)NO complexes were obtained using laser powers of 1 to 2 mW (measured at the sample) and a reciprocating translation stage. Spectra were recorded over periods of 1 to 10 min, and Raman shifts (±1 cm^−1^) were calibrated relative to indene and CD_3_CN standards.

### Kinetics measurements

Prior to kinetic measurements of Fe(II)XO complexes, solutions of Fe(II) McCP-β, were prepared anaerobically by reducing as-isolated Fe(III) protein with excess dithionite, followed by removal of reductant using a minispin desalting column (Zeba filter; Pierce). Kinetic measurements of Fe(II)CO and Fe(II)O_2_ McCP-β complexes (pH 8.9, 25 °C) were performed using an Applied Photophysics SX.18 MV-R stopped-flow spectrophotometer (dead time ∼1.5 ms) housed within an anaerobic vacuum atmosphere glove box. Stopped-flow measurements of CO release were initiated by mixing a solution of Fe(II)CO complex with a 1:1 ratio of buffer containing excess NO (0.45–0.9 mM after mixing) and monitoring the increase in Fe(II)NO absorption of monochromatic light at ∼385 nm using a photomultiplier detector. Values of *k*_off_, determined from single exponential fits of 385 nm time courses, are the average of three to five experiments. Transient Fe(II)O_2_ complexes of Fe(II) McCP-β were characterized by rapidly mixing solutions of Fe(II) protein with equal volumes of buffer containing dissolved O_2_ (32–650 μM after mixing) and monitoring the resultant absorbance changes over timescales of 1.0 to 15 s using a photodiode array detector. Kinetic measurements of Fe(II)NO formation were performed using an Applied Photophysics SX-20 stopped-flow spectrophotometer (dead time ∼1.5 ms) (25 °C) at pH 5, 7.5, and 9. Reactions were initiated by mixing a solution of Fe(II) protein (10 μM in heme) with a 1:1 ratio of anaerobic buffer containing the NO donor, PROLI NONOate, to acquire the desired NO concentrations in the range of 50 to 5000 μM before mixing. The concentrations of dissolved NO were maintained at a ∼10-fold excess over the heme-binding sites (5 μM after mixing) to ensure pseudo–first-order conditions. Reactions were monitored with monochromatic light at 395 and 417 nm using a photomultiplier detector as well as with broad band light (300–700 nm) using a photodiode array detector. Pseudo–first-order rate constants at each concentration of NO were determined by fitting exponential time courses using a least-squares fitting method. Measurements of Fe(II)NO *k*_off_ values (25 °C) were performed anaerobically on samples housed within anaerobic septum-sealed quartz cuvettes, using a Cary 60 UV–visible scanning spectrophotometer with a temperature-controlled cell holder. Release of NO from Fe(II)NO complexes was initiated by adding a small volume (∼10 μl) of concentrated Fe(II)NO complex to an anaerobic cuvette containing a buffered solution of sodium dithionite (0.65–60 mM) as NO scavenger and excess CO (0.50–1.0 mM). Immediately upon mixing, the rate of Fe(II)NO→Fe(II)CO conversion was monitored *via* time-resolved UV–visible absorption spectra (360–440 nm) recorded at intervals of 15 s. Values of *k*_off_ were determined from single exponential fits of the 418 nm time course (corresponding to 6cCO formation) and represent the average of three to five experiments.

## Data availability

Coordinates and structure factors for all structures are deposited in the Protein Data Bank with accession codes listed in the relevant tables. The data in the article are available from the authors upon request.

## Supporting information

This article contains supporting information (4).

## Conflict of interest

The authors declare that they have no conflicts of interest with the contents of this article.
