# Supplementary material for: A heme pocket aromatic quadrupole modulates gas binding to cytochrome c′-β: Implications for NO sensors
Source: J Biol Chem. 2023 Apr 24;299(6):104742. doi: 10.1016/j.jbc.2023.104742 (PMC10318465; doi:10.1016/j.jbc.2023.104742)
Supplement: Supporting information [file mmc1.docx]

**SUPPORTING INFORMATION**

Table S1 **Data collection, processing and refinement statistics**

| Dataset | McCP Ferrous | As Isolated F32V | F32V Ferrous | As Isolated F61V | F61V Ferrous |
| --- | --- | --- | --- | --- | --- |
| Resolution (Å) | 37.35-1.68 | 74.71-1.75 | 52.96-1.74 | 74.57-1.96 | 47.2-2.04 |
| Unit cell, (Å) | a=b=c=  105.56 | a=b=c=  105.67 | a=b=c=  105.80 | a=b=c=  105.47 | a=b=c=  105.54 |
| Unique reflections | 44814 (2216) | 40548 (2016) | 39950 (2001) | 28359 (2087) | 24699 (1706) |
| Completeness (%) | 100 (100) | 100 (100) | 99.7 (100) | 100 (100) | 97.5 (100) |
| Redundancy | 6.7 (6.8) | 6.6 (6.8) | 6.7 (6.8) | 8.2 (10.3) | 2.5 (2.6) |
| R_meas_ | 0.048 (1.42) | 0.074 (2.28) | 0.062 (2.34) | 0.075 (2.03) | 0.064 (0.86) |
| I/σ(I) | 24.2 (1.5) | 17.2 (0.6) | 19.4 (0.7) | 14.9 (1.1) | 23.3 (1.3) |
| CC_1/2_ | 1.0 (0.5) | 1.0 (0.3) | 1.2 (0.03) | 1.0 (0.5) | 1.0 (0.6) |
| WilsonB-factor (Å^2^) | 28.0 | 28.9 | 32.2 | 45.7 | 42.9 |
| R_work_ | 0.192 | 0.186 | 0.182 | 0.187 | 0.184 |
| R_free_ | 0.228 | 0.213 | 0.209 | 0.210 | 0.215 |
| RMSD bond length (°) | 0.0147 | 0.0120 | 0.0156 | 0.0115 | 0.0116 |
| RMSD bond angles (Å) | 2.322 | 2.094 | 2.163 | 2.060 | 2.279 |
| Ramanchandran favoured (%) | 95.90 | 95.90 | 95.90 | 95.15 | 94.78 |
| PDB accession code | 7ZVZ | 7ZS4 | 7ZSV | 7ZRW | 7ZRX |

**
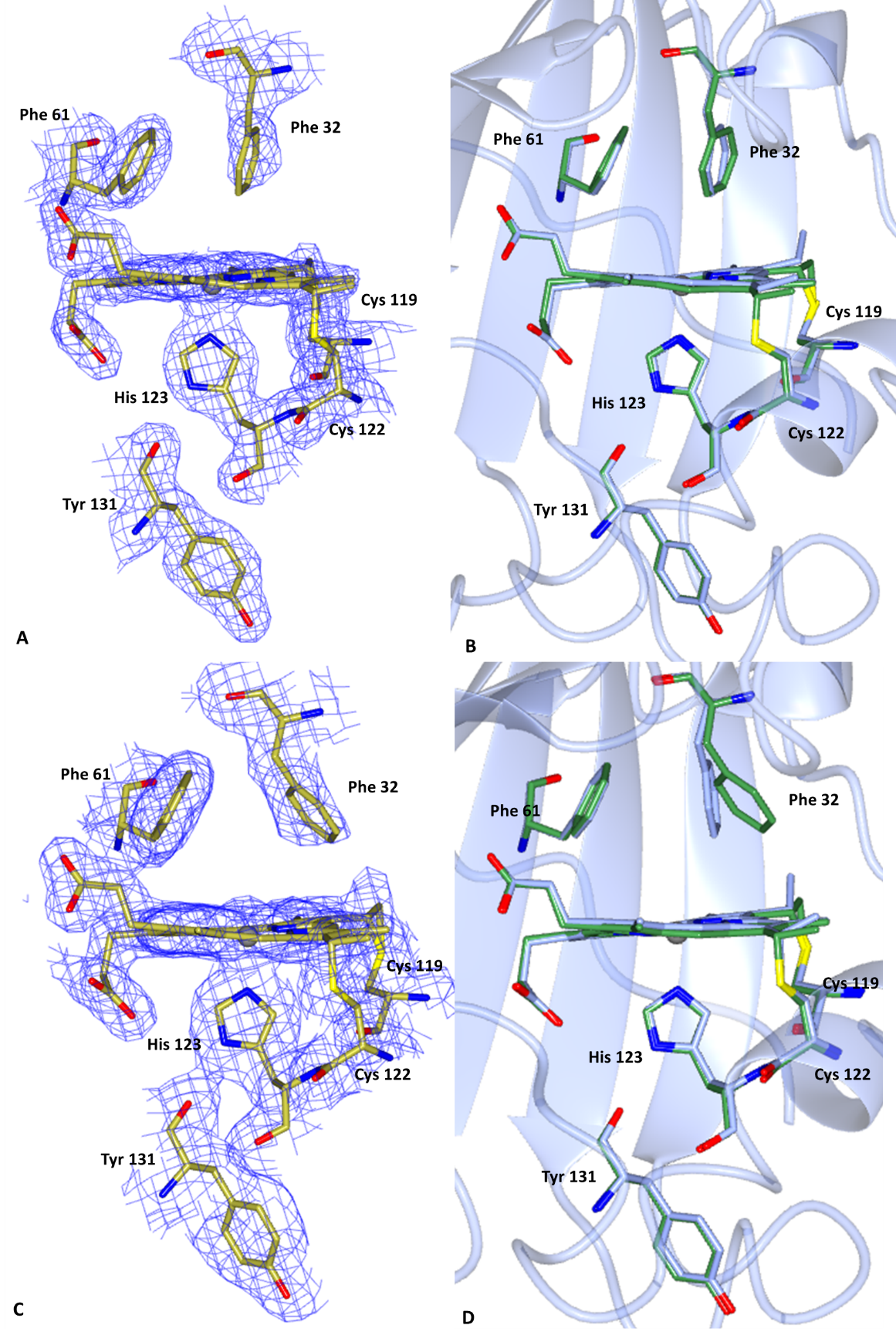
**

**Figure S1.** Superposition of the chemically reduced Fe(II) ligand-free heme structure of wt McCP-β with that of previously reported as-isolated McCP-β crystallised from the Fe(III) state ^4^. Heme A is shown in panels A and B and heme B is shown in panels C and D. Reducing wt McCP-β causes a slight movement and rotation in Phe32 compared to the as isolated structure, however this movement/rotation is less than that observed upon gas binding (Figure 1).

**Figure S2.** RR spectra of McCP-β complexes: porphyrin marker region (left panel) and low frequency region (right panel). Red traces are those of the Fe(II)CO complex at pH 7. Blue traces correspond to the pH-dependent coordination of the Fe(II)NO complex: pH 10 (6cNO), pH 7 (6cNO/5cNO mixture) and pH 4 (5cNO). Spectra were recorded at room temperature with 407-nm excitation. Asterisks denote RR bands attributed to a minor 5cFe(II) component. The 1360 cm^-1^ band (labelled †) apparent in the RR spectrum of the Fe(II)NO complex at pH 4 is attributed to the ν_4_ mode of a 4cFe(II) photoproduct.

**Figure S3**. Upper panel: pH-dependent UV-visible absorption spectra of the Fe(II)NO complex of wt McCP-β. Lower panel: the fraction of protein in the 5cNO form as a function of pH (see Materials and Methods). A fit to a single proton transition yields a p*K*_a_ value of 7.17 ± 0.03.


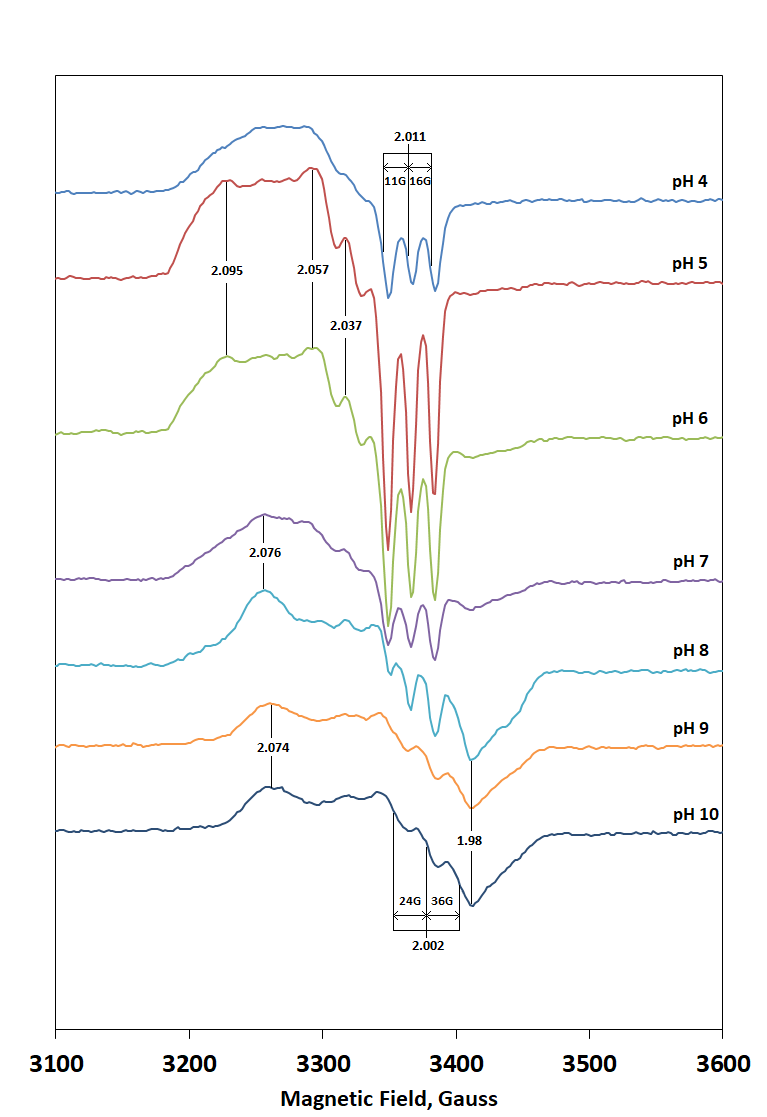


**Figure S4**. pH dependence of the X-band EPR spectra at 10 K of NO bound McCP-β. Low-temperature (10 K) EPR spectra of the Fe(II)NO complex shows a pH-dependent equilibrium, with a typical 5cNO 3-line hyperfine pattern seen at pH 4 – 6, shifting to an unresolved 9-line hyperfine pattern associated with 6cNO geometry at pH 9 – 10. The line shape at pH 7 and 8 suggests a mixture of 6cNO and 5cNO species.

**Figure S5.** Top panel: Determination of *k*_off_(NO) for the wt McCP-β Fe(II)NO complex (pH 9.5, 25 °C). Upon reaction with NO scavenger (44 mM dithionite) and CO (0.5 mM), the Fe(II)NO complex converts to the Fe(II)CO complex with NO release as the rate-determining step. The value of *k*_off_ (0.011 ± 0.001 s^-1^) was obtained from a 1 exp fit (red trace) of the 418 nm time course. Bottom panel: stopped-flow measurement of *k*_off_(CO) for the wt McCP-β Fe(II)CO complex (pH 7.0, 25 °C). Upon addition of excess NO (0.95 mM), the Fe(II)CO complex (blue trace) converts to the Fe(II)NO complex (magenta trace) with CO release as the rate-determining step. The value of *k*_off_ (0.202 ± 0.001 s^-1^) was obtained from a 1 exp fit (inset, red trace) of the 385 nm time course (inset, black trace).

**
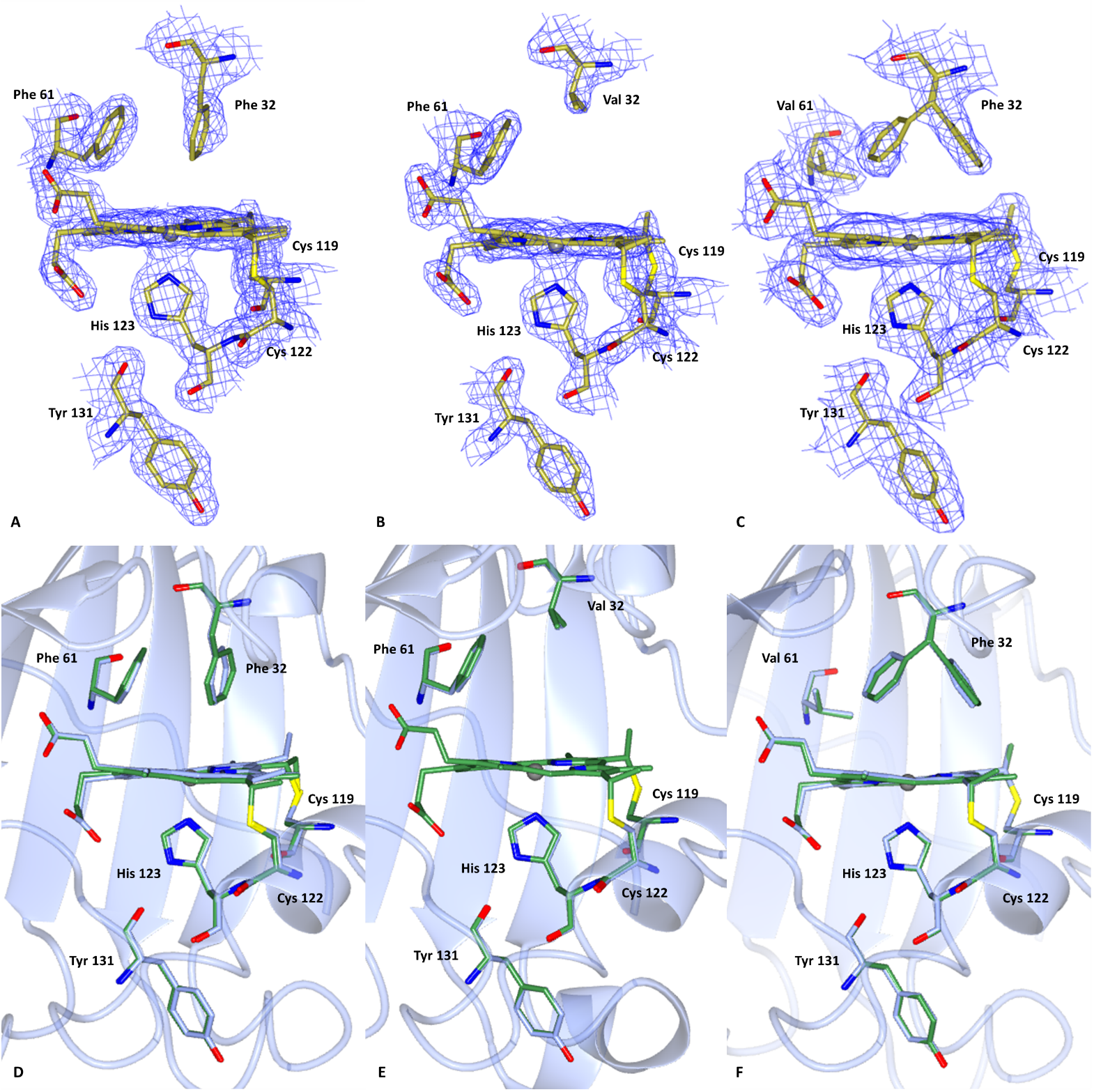
**

**Figure S6.** Ligand free Fe(II) forms of wt McCP-β (A), F32V (B) and F61V (C), superimposition of hemes A (blue) and B (green) of wt McCP-β (D), F32V (E) and F61V (F). Upon mutation of Phe 32 the only change to the heme pocket is that of the mutation, which sits in a relatively similar position to the Phe residue it has replaced (B,E). All other residues remain in the same position as seen in the Fe(II) WT McCP structure. Mutation of Phe 61 allows two conformations of Phe 32 to be present in both heme A and B (C,F). The positioning and orientation of these both differ from what can be seen in the Fe(II) WT McCP crystal structure (A,D)


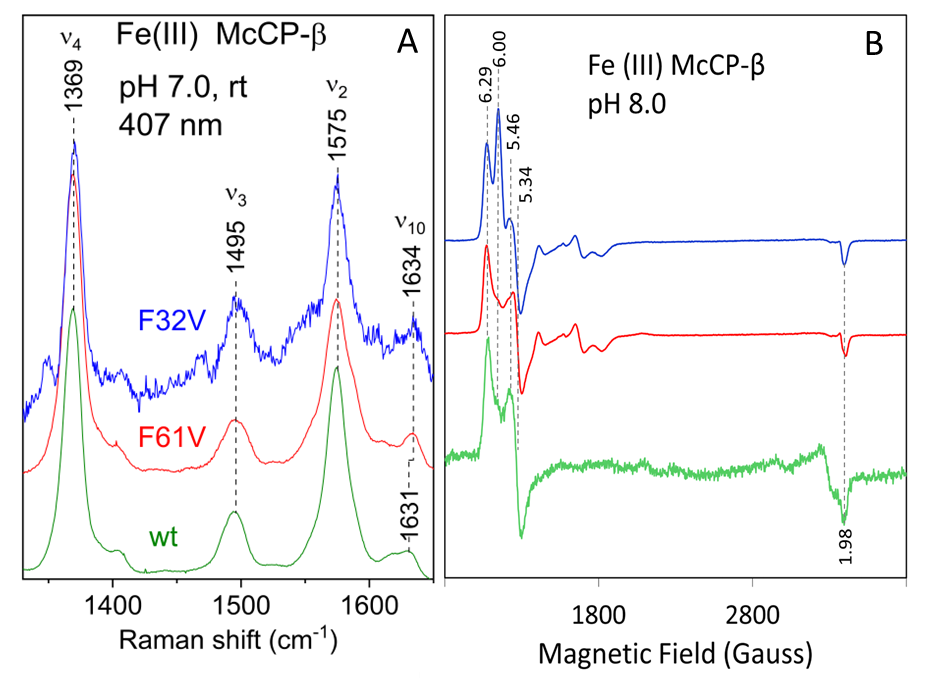


**Figure S7.** Room-temperature RR spectra (A) and low-temperature (10 K) EPR spectra (B) of Fe(III) as-isolated forms of McCP-β. Data for the F61V and F32V variants obtained in the present study are shown along with previous data for wt protein. ^4^ Porphyrin marker RR frequencies for F32V and F61V variants are similar to those of wt McCP-β, and support the presence of 5cHS Fe(III) heme with no distal solvent ligand. As previously reported for wt McCP-β,-EPR spectra of F32V and F61V variants show a mixture of two high spin Fe(III) species with different rhombicities Additional signals in the F32V and F61V EPR spectra are attributed to a minor population of free heme.

**Figure S8.** Low-frequency room temperature RR spectra of 6cCO complexes of wt, F61V, and F32V McCP-β prepared with ^12^CO (blue traces) and ^13^CO (magenta traces). The wavenumbers and isotope shifts of RR bands assigned to ν(Fe–CO) modes are indicated in red.

**Figure S9.** Determination of *k*_off(CO)_ values for Fe(II)CO complexes of F61V McCP-β (upper panel) and F32V McCP-β (lower panel) by stopped-flow UV-visible absorption spectroscopy at pH 7.0. After mixing with 1.0 mM NO, the Fe(II)CO complexes (black traces) convert to Fe(II)NO end products (red traces). Insets show associated time courses and single exponential fits for Fe(II)CO → Fe(II)NO conversion, yielding a *k*_off(CO)_ values of 0.32 (± 0.01) s^-1^ (F61V) and 0.13 (± 0.01) s^-1^ (F32V).


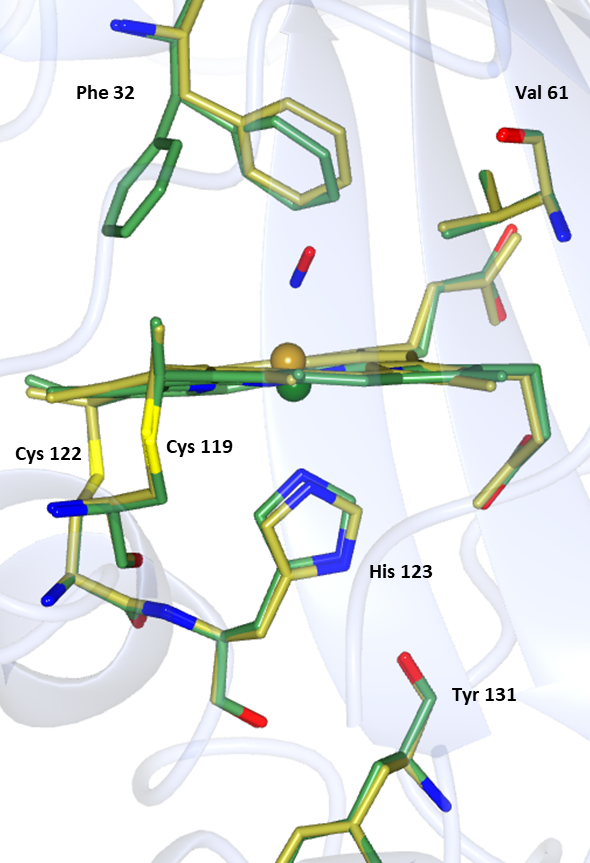


**Figure S10**. F61V Fe(II)NO crystal structure (gold) compared to the F61V Fe(II) structure (green) clearly shows the Fe atom of the heme has moved upwards along with a slight movement downwards of His123. These together cause an elongation of the Fe-His distance (2.93/3.03 Å in comparison to 2.18/2.20 Å in wt McCP) which may be leading to partial dissociation of the His to the heme.

**Figure S11.** pH-dependent UV-visible absorption spectra of the Fe(II)NO complexes of wt McCP-β (upper panel), F61V McCP-β (middle panel), and F32V McCP-β (lower panel).


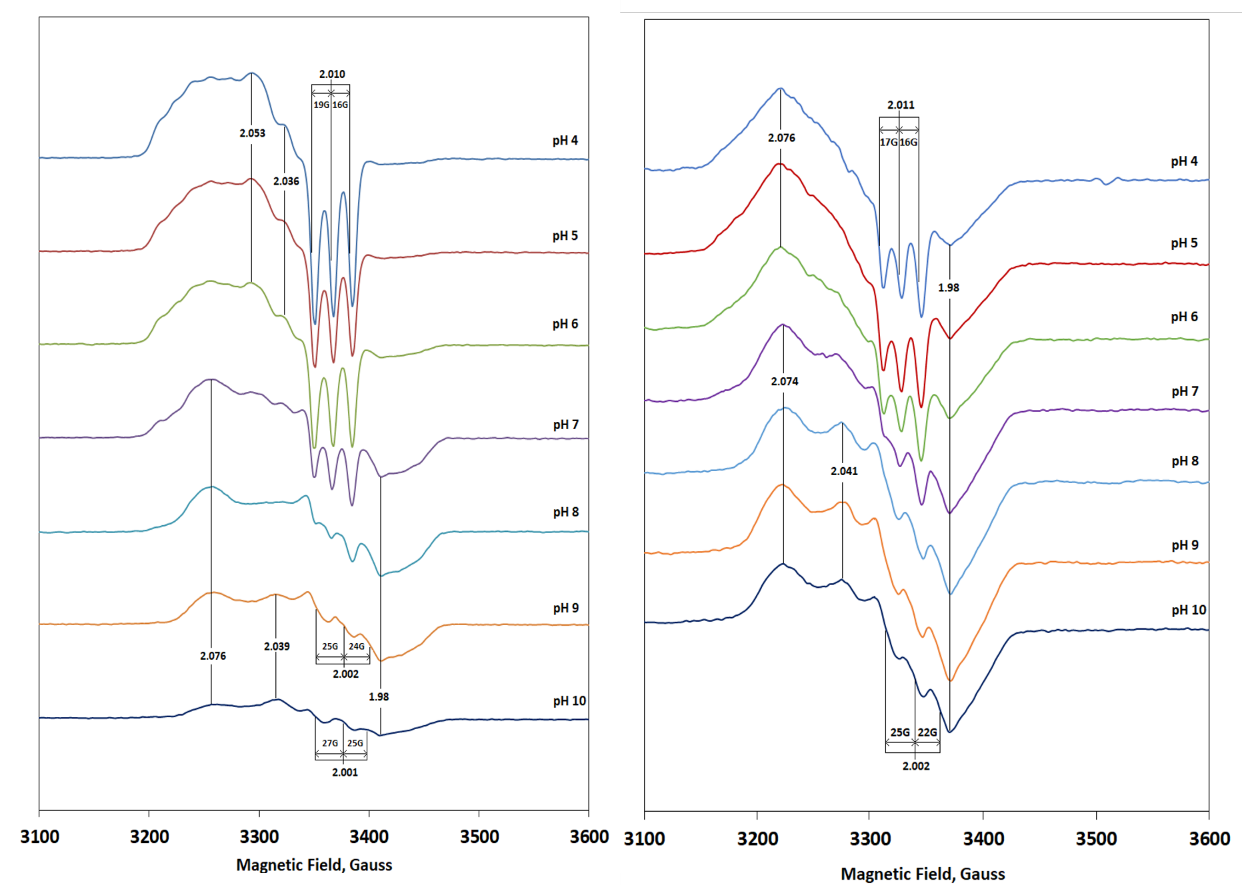


**Figure S12**. Left panel: pH dependence of the X-band EPR spectra at 10 K of NO bound F61V variant. Low-temperature (10 K) EPR spectra of the Fe(II)NO complex shows a pH-dependent equilibrium, with a typical 5cNO 3-line hyperfine pattern seen at pH 4 – 6, shifting to an unresolved 9-line hyperfine pattern associated with 6cNO geometry at pH 9 – 10. The line shape at pH 7 and 8 suggests a mixture of 6cNO and 5cNO species. Right panel: pH dependence of the X-band EPR spectra at 10 K of NO bound F32V variant. Low-temperature (10 K). Compared to the F61V variant, a lower pH is required to achieve a predominantly 5cNO 3-line hyperfine pattern.

**Figure S13.** Determination of the *k*_off(NO)_ values for the 6c Fe(II)NO complexes of F61V McCP-β (upper panel) and F32V McCP-β (lower panel) by time-resolved UV-visible absorption spectroscopy at pH 9.5. After manually mixing the 6cNO complexes with buffer containing 1.0 mM CO and excess sodium dithionite, the Fe(II)NO complexes convert to a Fe(II)CO end product with NO release as the rate-determining step. Single exponential fits of time courses and at 418 nm yield *k*_off(NO)_ values of 0.016 (± 0.001) s^-1^ (F61V) and 0.0045 (± 0.0001) s^-1^ (F32V).
